# Bruceantin targets HSP90 to overcome resistance to hormone therapy in castration-resistant prostate cancer

**DOI:** 10.7150/thno.51478

**Published:** 2021-01-01

**Authors:** Sue Jin Moon, Byong Chang Jeong, Hwa Jin Kim, Joung Eun Lim, Hye-Jeong Kim, Ghee Young Kwon, Joshua A. Jackman, Jeong Hoon Kim

**Affiliations:** 1 Department of Health Sciences and Technology, Samsung Advanced Institute for Health Sciences and Technology, Sungkyunkwan University, Seoul, 06351, Korea; 2 Department of Biomedical Sciences, Samsung Biomedical Research Institute, Samsung Medical Center, Seoul, 06351, Korea; 3 Department of Urology, Samsung Medical Center, Sungkyunkwan University School of Medicine, Seoul, 06351, Korea; 4 C&C Research Laboratories, Sungkyunkwan University, Suwon, 16419, Korea; 5 Department of Pathology, Samsung Medical Center, Sungkyunkwan University School of Medicine, Seoul, 06351, Korea; 6 School of Chemical Engineering and Biomedical Institute for Convergence at SKKU (BICS), Sungkyunkwan University, Suwon, 16419, Korea

**Keywords:** castration-resistant prostate cancer, androgen receptor, androgen receptor variant 7, bruceantin, HSP90

## Abstract

**Rationale:** Aberrant androgen receptor (AR) signaling *via* full-length AR (AR-FL) and constitutively active AR variant 7 (AR-V7) plays a key role in the development of castration-resistant prostate cancer (CRPC) and resistance to hormone therapies. Simultaneous targeting of AR-FL and AR-V7 may be a promising strategy to overcome resistance to hormone therapy. This study aimed to identify novel drug candidates co-targeting AR-FL and AR-V7 activities and elucidate their molecular mechanism of anti-CRPC activities.

**Methods:** Using a CRPC cell-based reporter assay system, we screened a small library of antimalarial agents to explore the possibility of repositioning them for CRPC treatment and identified bruceantin (BCT) as a potent anti-CRPC drug candidate. A series of cell-based, molecular, biochemical, and in vivo approaches were performed to evaluate the therapeutic potential and molecular mechanism of BCT in CRPC. These approaches include reporter gene assays, cell proliferation, RNA-seq, qRT-PCR, mouse xenografts, co-immunoprecipitation, GST pull-down, immobilized BCT pull-down, molecular modeling, and bioinformatic analyses.

**Results:** We identified BCT as a highly potent inhibitor co-targeting AR-FL and AR-V7 activity. BCT inhibits the transcriptional activity of AR-FL/AR-V7 and downregulates their target genes in CRPC cells. In addition, BCT efficiently suppresses tumor growth and metastasis of CRPC cells. Mechanistically, BCT disrupts the interaction of HSP90 with AR-FL/AR-V7 by directly binding to HSP90 and inhibits HSP90 chaperone function, leading to degradation of AR-FL/AR-V7 through the ubiquitin-proteasome system. Clinically, HSP90 expression is upregulated and correlated with AR/AR-V7 levels in CRPC.

**Conclusion:** Our findings suggest that BCT could serve as a promising therapeutic candidate against CRPC and highlight the potential benefit of targeting AR-FL/AR-V7-HSP90 axis to overcome resistance caused by aberrant AR-FL/AR-V7 signaling.

## Introduction

Androgen receptor (AR), an androgen-activated transcription factor, plays a pivotal role not only in normal development and growth of the prostate but also in prostate cancer (PCa) development and progression to castration-resistant prostate cancer (CRPC) 1-3. Targeting of full-length AR (AR-FL) and androgen synthesis is a mainstay of therapy for PCa patients. Although PCa initially responds to hormone therapies such as androgen biosynthesis inhibitors (*e.g.,* abiraterone [ABI]) and anti-androgens (*e.g.,* enzalutamide [ENZ] and bicalutamide [BIC]), PCa eventually progresses to advanced metastatic CRPC, which is resistant to conventional androgen deprivation and anti-androgen therapies 1-5. Accumulating evidence indicates that alternatively spliced AR variants (AR-Vs) play critical roles in promoting resistance to ABI and ENZ therapies and in driving metastatic CRPC progression 1-3, 5.

To date, over 20 AR-Vs have been identified in clinical specimens and PCa cell lines 2, 6. Among them, AR-V7 is the most abundant and frequently found variant in PCa cell lines and CRPC tissues 1, 2, 7. AR-V7 is a truncated form of AR lacking the C-terminal ligand-binding domain (LBD) which is the direct or indirect target of ENZ and ABI, respectively, but contains intact AR N-terminal domain (NTD) and DNA-binding domain followed by 16 unique amino acids from a cryptic exon 3b 1, 2, 5-7. AR-V7 functions as a ligand-independent constitutively active transcription factor that induces castration-resistant and anti-androgen-resistant PCa growth in vitro and in vivo. Furthermore, AR-V7 expression in tumor tissues and circulating tumor cells of CRPC patients correlates with poor prognosis, shorter progression-free survival and overall survival, and resistance to secondary hormone therapy 8, 9. In addition, AR-FL and AR-V7 form heterodimers, and their binding to chromatin is interdependent 10, 11. Thus, it is urgent and critical to develop novel therapeutic strategies co-targeting AR-FL and AR-V7 for CRPC treatment.

Heat shock protein 90 (HSP90) is an ATP-dependent molecular chaperone and forms a dynamic chaperone complex with HSP70 and co-chaperones to regulate the folding and stability of more than 200 client proteins including AR 12-16. Cancer cells depend on HSP90 to maintain the stability and function of oncoproteins and to buffer cellular stresses caused by malignant transformation 12, 13, 16. In PCa cells, HSP90 positively regulates the stability and activity of AR, and HSP90 inhibitors induce AR degradation and apoptosis 12, 13. Thus, targeting HSP90 might provide a beneficial therapeutic strategy to treat androgen-dependent PCa and androgen-independent CRPC.

Recent studies reported that ailanthone, artesunate, and chloroquine have anticancer activity against CRPC cells 17-19. Based on the fact that they are antimalarial agents, we screened a small library of antimalarial agents to explore the possibility of repositioning antimalarials for CRPC treatment and identified bruceantin (BCT) as a potent inhibitor targeting both AR-FL and AR-V7. We found that BCT inhibits the transcriptional activities of AR-FL/AR-V7 and suppresses in vivo growth and metastatic potential of CRPC cells. Mechanistically, BCT disrupts the interaction of HSP90 with AR-FL/AR-V7 by directly binding to and inhibiting HSP90 chaperone function, leading to enhanced degradation of AR-FL/AR-V7 through the ubiquitin-proteasome system. Collectively, our findings suggest that BCT could serve as a promising therapeutic candidate against CRPC.

## Materials and Methods

### Cell culture

All cell lines used, except C4-2B-MDVR (an ENZ-resistant C4-2B cell line), were purchased from American Type Culture Collection (ATCC) or Korean Cell Line Bank (KCLB). C4-2B-MDVR cells were kindly provided by Dr. Christopher Evans (UC Davis). Cells were tested regularly for mycoplasma contamination by qPCR and authenticated by STR analysis. 22RV1, C4-2B, C4-2B-MDVR, and LNCaP were grown in RPMI 1640 supplemented with 10% fetal bovine serum (FBS). DU145, PC3, VCaP, and 293T cells were cultured in DMEM with 10% FBS. RWPE-1 cells were cultured in KSFM containing 5 ng/mL of EGF and 25 μg/mL of bovine pituitary extract. For hormone starvation, cells were cultured in RPMI supplemented with 5% dextran-coated charcoal (DCC)-treated FBS for 72 h prior to vehicle, dihydrotestosterone (DHT), or inhibitor treatment.

### Screening of inhibitors against transcriptional activities of AR-FL and AR-V7

22RV1 cells cultured in DCC-FBS medium were transfected with MMTV-LUC and pRL-SV40 (Promega) using Lipofectamine 3000 (ThermoFisher Scientific) and plated into 96-well plates. After 48 h transfection, cells were treated with vehicle, 20 nM DHT, compounds (10, 1, or 0.1 μM), or 20 nM DHT with compounds for 24 h and then lysed for dual luciferase reporter assays (Promega). Compounds screened in this study are listed in Table S1.

### RNA sequencing data analysis

22RV1 cells were cultured in full medium (androgen-undepleted medium: RPMI 1640 supplemented with 10% FBS, which contains endogenous levels of androgen and growth factors) and treated with DMSO or 10 nM BCT for 24 h. Total RNAs were purified using RNeasy Plus Mini Kit (Qiagen). RNA-seq libraries from 1 μg of total RNA were prepared using TruSeq RNA Library Prep Kit v2 (Illumina) according to the manufacturer's instructions. The RNA-seq libraries were sequenced as 100 bp paired-end reads on an Illumina HiSeq 2500 with HiSeq PE Rapid Cluster Kit v2 and HiSeq Rapid SBS Kit v2 (Illumina), generating an average of ~120 million reads per sample. RNA-seq reads were filtered and trimmed using Trimmomatic and were aligned to the human reference genome (hg19/GRCh37) using RNA-seq read mapper HISAT2 (v2.1.0). Mapped reads were assembled using StringTie (v1.3.4d), and gene expression levels were calculated as fragments per kilobase of transcript per million mapped reads (FPKM). Differentially expressed genes were determined based on the false discovery rate (FDR) threshold 0.01 and log2 fold change cutoff of 0.85 and clustered with Hierarchical Clustering algorithm.

### Xenograft experiments

Mouse subcutaneous xenograft experiments were performed as described in our previous study 20, 21. Briefly, 3x10^6^ of 22RV1-LUC cells, which stably express luciferase and hygromycin-resistant genes, suspended in 100 μL Matrigel/PBS (50:50 mixture) were injected subcutaneously into the left flank of 6-week-old male athymic BALB/c nu/nu mice (Orient Bio, Korea). Once the tumor sized reaches 50 to 100 mm^3^, tumor-bearing mice were randomly assigned to two groups (control and BCT group, 10 mice per group) and treated intraperitoneally (once every three days) or orally (once every two days) with vehicle or BCT (1 mg/kg or 2 mg/kg body weight). For oral gavage administration, BCT was dissolved in 1% sodium carboxymethyl cellulose (Na-CMC) and 1% Tween 80 vehicle solution. Tumor volumes were measured twice a week in three dimensions using a caliper as described previously 21. For in vivo bioluminescence imaging, mice were anesthetized, and 3 mg of D-luciferin (Xenogen) was administered intraperitoneally 10 min before imaging. Bioluminescence was measured using the IVIS Spectrum Imaging System (Xenogen, PerkinElmer). At the end of the xenograft studies, tumor tissues were collected from euthanized mice. Half of the tissues were fixed in 4% formaldehyde for histological analysis, and the other half were frozen for further analysis. To derive highly metastatic cells, 1x10^6^ of 22RV1-LUC cells were subcutaneously injected into nude mice. After 3 weeks, subcutaneous 22RV1 xenograft tumors were harvested, washed with PBS containing 2x penicillin/streptomycin solution (Gibco), minced with sterile scalpels, and further digested with Liberase TM research grade (Sigma-Aldrich) for 1 h. The pure 22RV1-LUC cell population (22RV1-LUC-T1) was obtained by subculturing 5 times in the presence of hygromycin (200 μg/mL) to remove host cell contamination. For metastasis experiments, 22RV1-LUC-T1 cells (5x10^5^ cells) were injected into the left cardiac ventricle of 6-week-old male nude mice guided by ultrasound (VisualSonics Vevo 2100 imaging system). The mice were randomly split into two groups (control and BCT group, 8 mice per group) and treated orally once every two days with vehicle or BCT (2 mg/kg body weight). Bioluminescence imaging was performed weekly to monitor tumor metastasis. Animal experiments were conducted with the approval of the Institutional Animal Care and Use Committee of Laboratory Animal Research Center at Samsung Biomedical Research Institute.

### Pull-down assays using BCT-conjugated beads

Profinity epoxide resin (Bio-Rad) was swollen and washed in ddH_2_O and then resuspended in coupling buffer (0.1 N NaOH, pH 13.0). BCT (5 mg) was dissolved in coupling buffer and conjugated with Profinity epoxide resin at room temperature for 18 h. After washing with coupling buffer, remaining active epoxide groups were blocked with 1 M ethanolamine (pH 8.0) for 4 h. The BCT-conjugated beads were washed with coupling buffer, wash buffers (WB1, 100 mM acetate, 500 mM NaCl, pH 4.5; WB2, 100 mM Na_2_HPO_4_, 500 mM NaCl, pH 8.0), and PBS. The control unconjugated Profinity beads were prepared as described above without addition of BCT. The control or BCT-conjugated beads were incubated with cell lysates overnight and washed with FLAG lysis buffer. Bound proteins were detected with specific antibodies as indicated in figures.

### RNA interference

The depletion of HSP90 was performed by transfection of 22RV1 cells with either non-specific (NS) siRNA or siRNA against HSP90α/β using Lipofectamine 2000 (ThermoFisher Scientific). The siRNA sequences used are listed in Table S4.

### Molecular Modeling

Molecular modeling studies were performed using the Schrodinger Suite (Schrodinger Inc.). The X-ray crystal structure of bruceantin extracted from the Protein Data Bank (PDB code: 3G71.pdb) was processed for further docking studies using LigPrep module in OPLS_2005 force field with tautomer states. The protein structure of HSP90 (PDB code: 1BYQ.pdb) was optimized using the Protein Preparation Wizard module. The receptor grid box was generated using the Glide module followed by docking study with Standard Precision mode. The OPLS_2005 force field and all default parameters were applied in all procedures. Ribbon diagrams of BCT-bound HSP90 structure were generated using CCP4MG software (http://www.ccp4.ac.uk/MG/).

### Bioinformatics and statistical analysis

Publicly available datasets from Gene Expression Omnibus (GEO) (https://www.ncbi.nlm.nih.gov/geo) and Oncomine (http://www.oncomine.org) were analyzed for HSP90 and AR mRNA expression. Three PCa microarray datasets (Singh Prostate, Vanaja Prostate, and Welsh Prostate) from Oncomine were analyzed for HSP90 isoform (HSP90α/β) expression in normal prostate and PCa. Two PCa microarray datasets (GSE6919 and GSE35988) were analyzed using PRISM v5.0 to investigate mRNA levels of HSP90 isoforms and AR in normal prostate (or benign prostate hyperplasia), primary PCa, and metastatic PCa (or CRPC) tissues. The correlation between HSP90 isoforms and AR mRNA expression in GSE97284 dataset were analyzed using the R2 Genomics Analysis and Visualization Platform (http://r2.amc.nl). HSP90α/β mRNA expression in different Gleason score PCa was determined using Singh Prostate dataset from Oncomine. Box plot and volcano plot analyses were performed using R software package (https://www.r-project.org/). Statistical analyses were performed with PRISM v5.0. Data are statistically analyzed by unpaired or paired two-tailed Student's t-tests for two groups or one-way ANOVA for multiple comparisons followed by Tukey's post-hoc test.

## Results

### BCT inhibits the transcriptional activity of AR-FL/AR-V7 and growth of PCa cells

**Figure 1:**
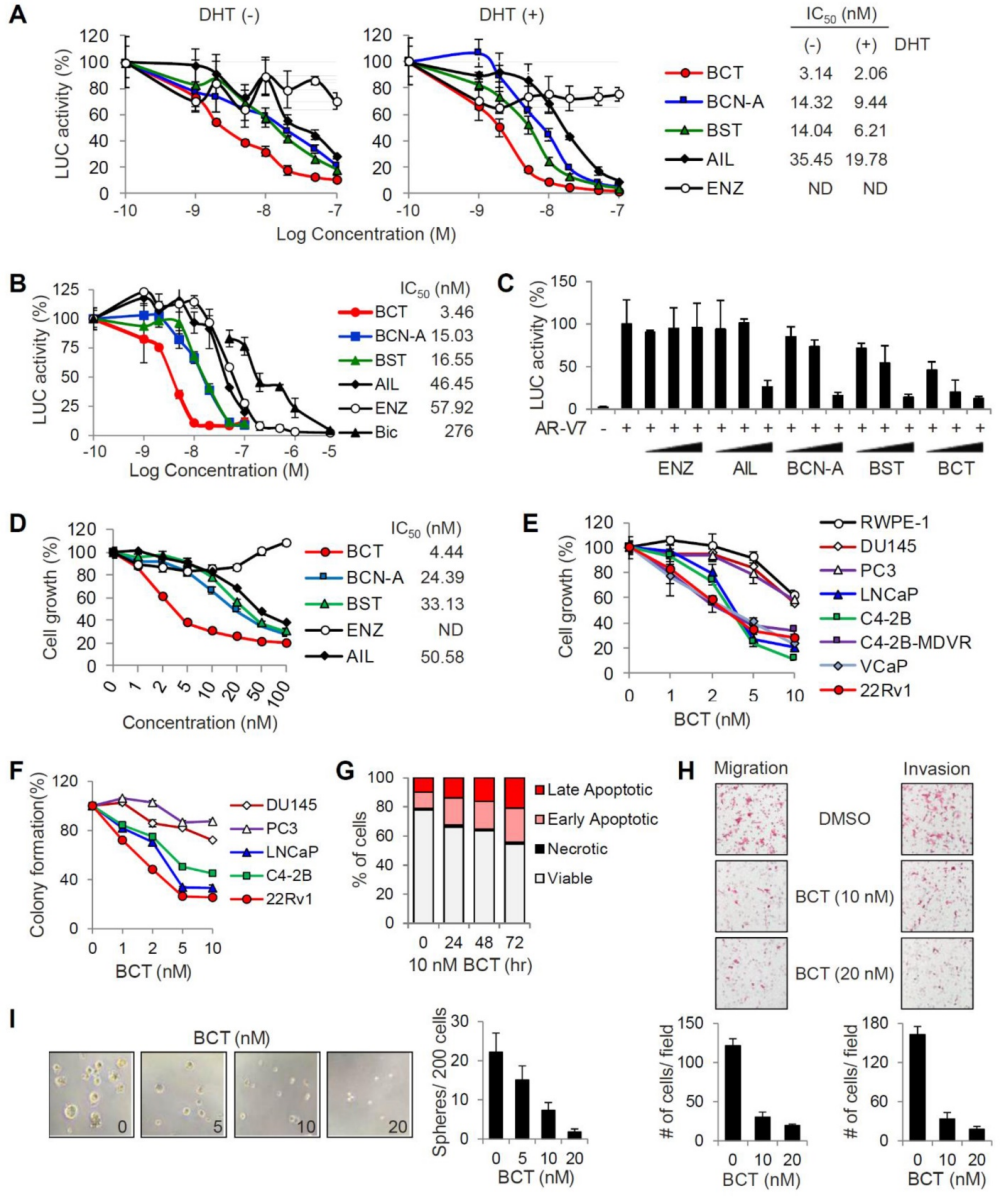
** Inhibitory effects of BCT on the transcriptional activity of AR-FL and AR-V7 and PCa cell proliferation.** (A) 22RV1 cells transfected with MMTV-LUC were treated with different concentrations of indicated compounds with or without DHT, luciferase activities were measured, and IC_50_ values were determined. Data are means ± s.d. (n = 3). (B, C) MMTV-LUC reporter assays in LNCaP with or without DHT (B) and AR-V7-transfected PC3 (C) were performed as described in (A). (D) 22RV1 cells were treated with different concentrations of indicated compounds. Cell proliferation was detected by MTT assays, and IC_50_ values were determined. ND, not determined. Data are means ± s.d. (n = 6). (E, F) Cell proliferation (E) and colony formation (F) assays of AR-positive and AR-negative PCa cells and normal prostate cells after treatment of various concentrations of BCT. Data are means ± s.d. (n = 6). (G) Cell cycle analysis of 22RV1 cells after BCT treatment. (H, I) Migration and invasion assays (H) and sphere formation assays (I) of 22RV1 cells after treatment of indicated concentrations of BCT.

To identify inhibitors targeting the transcriptional activities of both androgen-dependent AR-FL and androgen-independent, constitutively active AR-V7, we performed reporter assays to screen about 50 compounds which are selected based on their previously reported antimalarial activities (Table S1). CRPC 22RV1 cells, which express both AR-FL and AR-V7, were transfected with MMTV-LUC reporter and treated with each compound at an initial screening concentration of 10 μM in the presence or absence of DHT. After three rounds of screening with various concentrations (0.1, 1, and 10 μM) of each compound, we identified four quassinoids, BCT, brusatol (BST), bruceine A (BCN-A), and ailanthone (AIL), as the most potent inhibitors of AR-FL/AR-V7 transcriptional activities (Figure S1 and Table S2). To validate the inhibitory effects of individual quassinoids on AR-FL/AR-V7 activities, we repeated reporter assays in 22RV1 cells. All four quassinoids, but not the anti-androgen ENZ, efficiently inhibited DHT-independent (representing AR-V7 activity) and DHT-dependent (representing AR-FL activity) reporter activities with low nanomolar IC_50_ values (Figure 1A). Similarly, DHT-induced AR-FL activity was also strongly suppressed by four quassinoids, compared to ENZ and BIC, in LNCaP cells, (Figure 1B). In addition, while ectopically expressed AR-V7 activity was resistant to ENZ in PC3 cells, its transcriptional activity was also inhibited by four quassinoids in a dose-dependent manner (Figure 1C).

We next examined the effect of four quassinoids on the proliferation of ENZ-resistant 22RV1 cells. All the quassinoids tested, but not ENZ, potently inhibited 22RV1 cell proliferation in a dose-dependent manner, and BCT had the most potent growth inhibitory activity (IC_50_ = 4.44 nM) (Figure 1D). Given our initial results showing that BCT displayed the most potent inhibitory effects on AR-FL/AR-V7 activities and on cell growth of CRPC cells, we focused on BCT for further analysis. We confirmed that BCT potently inhibited the growth of AR-positive PCa cells, LNCaP and C4-2B, and AR-FL/AR-V7-positive CRPC cells, C4-2B-MDVR, VCaP, and 22RV1 (Figure 1E). Interestingly, BCT showed greater anti-proliferative and anti-clonogenic effects on AR-positive PCa cells than on normal prostate epithelial cells (RWPE-1) and AR-negative PCa cells (DU145 and PC3), which express no AR-FL/AR-V7, suggesting that, consistent with the results of reporter gene assays, BCT may specifically target AR-FL/AR-V7 signaling in AR-positive PCa cells (Figure 1E-F and Figure S2A). In addition, BCT induced apoptosis in 22RV1 cells in a time-dependent manner (Figure 1G and Figure S2B). BCT also strongly suppressed the migration and invasion of 22RV1 cells (Figure 1H). Moreover, BCT reduced the size and number of prostaspheres in sphere formation assays using 22RV1 cells (Figure 1I), indicating that BCT can inhibit the self-renewal of stem-like CRPC cells. Collectively, these results revealed that BCT could potently inhibit the growth of AR-positive PCa cells (including both androgen-sensitive and CRPC cells) with less effect on normal prostate cells and AR-negative PCa cells and suppress in vitro metastatic properties of CRPC cells.

### BCT inhibits AR-FL/AR-V7 signaling in CRPC cells

**Figure 2:**
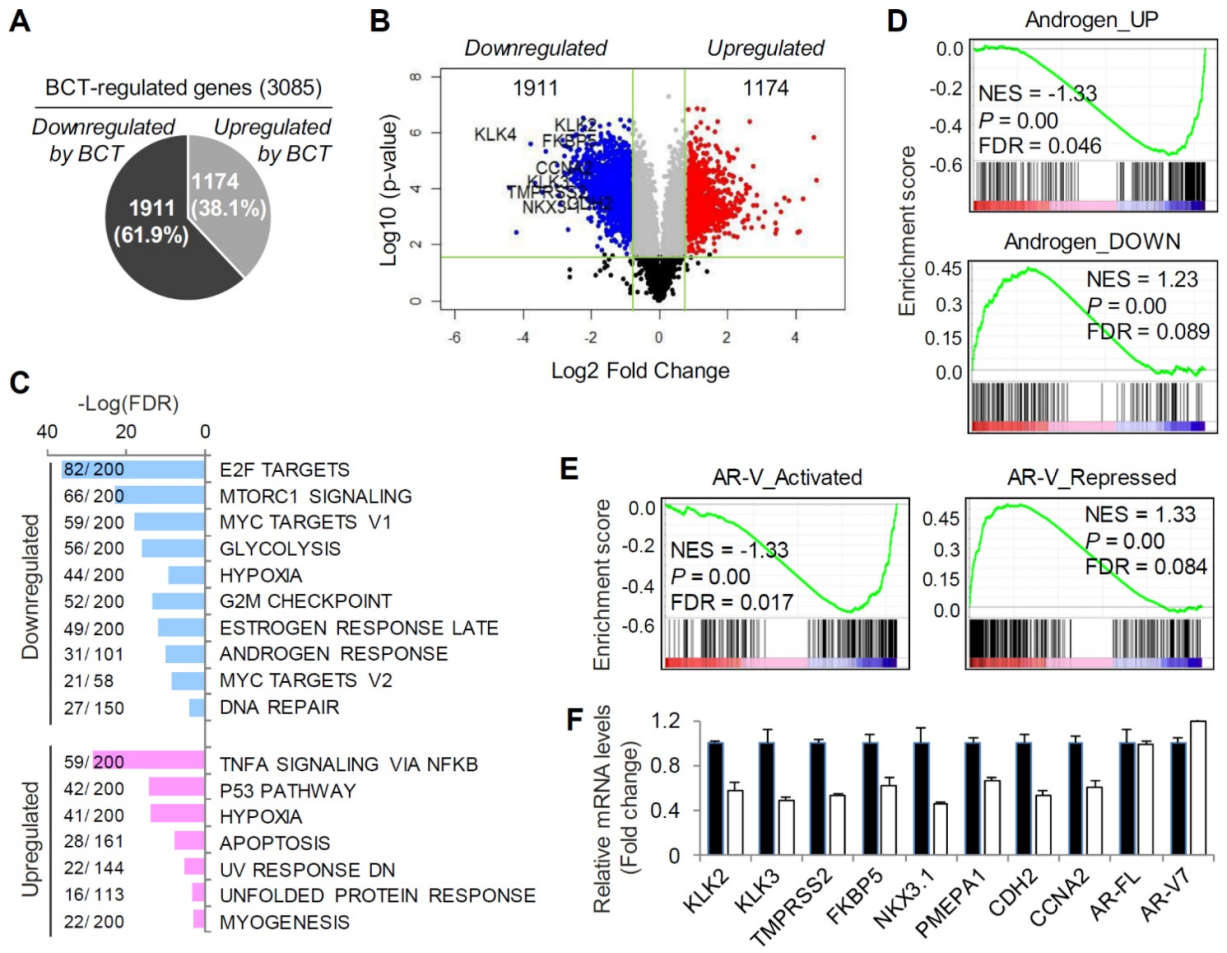
** BCT inhibits AR/AR-V7 signaling in CRPC cells.** (A, B) RNA-seq analysis of differential gene expression in 22RV1 cells cultured in full medium and treated with BCT. Pie (A) and volcano (B) plots show that 3085 genes are differentially expressed by BCT. (C) GSEA of top enriched Hallmark gene sets in 22RV1 cells treated by BCT. The number of genes identified in RNA-seq and the number of total genes curated for the specific term are shown as numerator and denominator, respectively. (D, E) GSEA of the AR-FL (D) and AR-V (E) gene signatures in 22RV1 cells treated with BCT. (F) Validation of BCT-regulated AR-FL/AR-V7 target genes by qRT-PCR in 22RV1 cells cultured in full medium. Data are expressed as fold change relative to control DMSO and are means ± s.d. (n = 3).

To identify gene expression programs that are regulated by BCT and to assess the effect of BCT on the expression of AR-FL/AR-V7-regulated transcriptome, we performed RNA-sequencing (RNA-seq) analyses using 22RV1 cells treated with or without BCT. RNA-seq analysis showed that 3085 genes were differentially expressed (log2FC > 0.85, FDR < 0.05) by BCT treatment, with 1911 downregulated and 1174 upregulated genes (Figure 2A-B and Figure S3A). Gene Set Enrichment Analysis (GSEA) using the Hallmark gene sets revealed that pathways involved in cancer initiation and progression (*e.g.*, E2F, mTORC1, Myc, and androgen response signalings) are downregulated by BCT (Figure 2C).

In contrast, the BCT-upregulated pathways include pathways related to cell death and apoptosis (*e.g.*, p53 pathway and apoptosis) and unfolded protein response. Further GSEA using the androgen target gene set revealed significant repression in the expression of androgen-upregulated genes and significant activation in androgen-downregulated gene expression (Figure 2D and Figure S3B). Similar results were observed in GSEA using AR-V-activated and repressed gene sets (Figure 2E). These results are consistent with our initial finding that BCT can inhibit the transcriptional activity of both AR-FL and AR-V7 and suggest the potential therapeutic use of BCT in ENZ-resistant CRPC. The BCT-downregulated genes include well-characterized AR-FL/AR-V7 target genes, such as KLK2, KLK3, TMPRSS2, NKX3.1, PMEPA1, and FKBP5, and AR-V7 unique target genes, such as CDH2 and CCNA2 (Figure 2B and Figure S3A-B). We validated their mRNA expression levels by qRT-PCR in full medium and confirmed the downregulation of AR-FL/AR-V7 target gene expression by BCT (Figure 2F). Similar results were obtained with 22RV1 cells, which were cultured in androgen-depleted medium and treated with BCT in the presence or absence of DHT (Figure S3C-D). Together, these results suggest an inhibitory role of BCT in AR-FL and AR-V7-mediated transcription in CRPC cells.

### BCT inhibits CRPC tumor growth and metastasis in vivo

**Figure 3:**
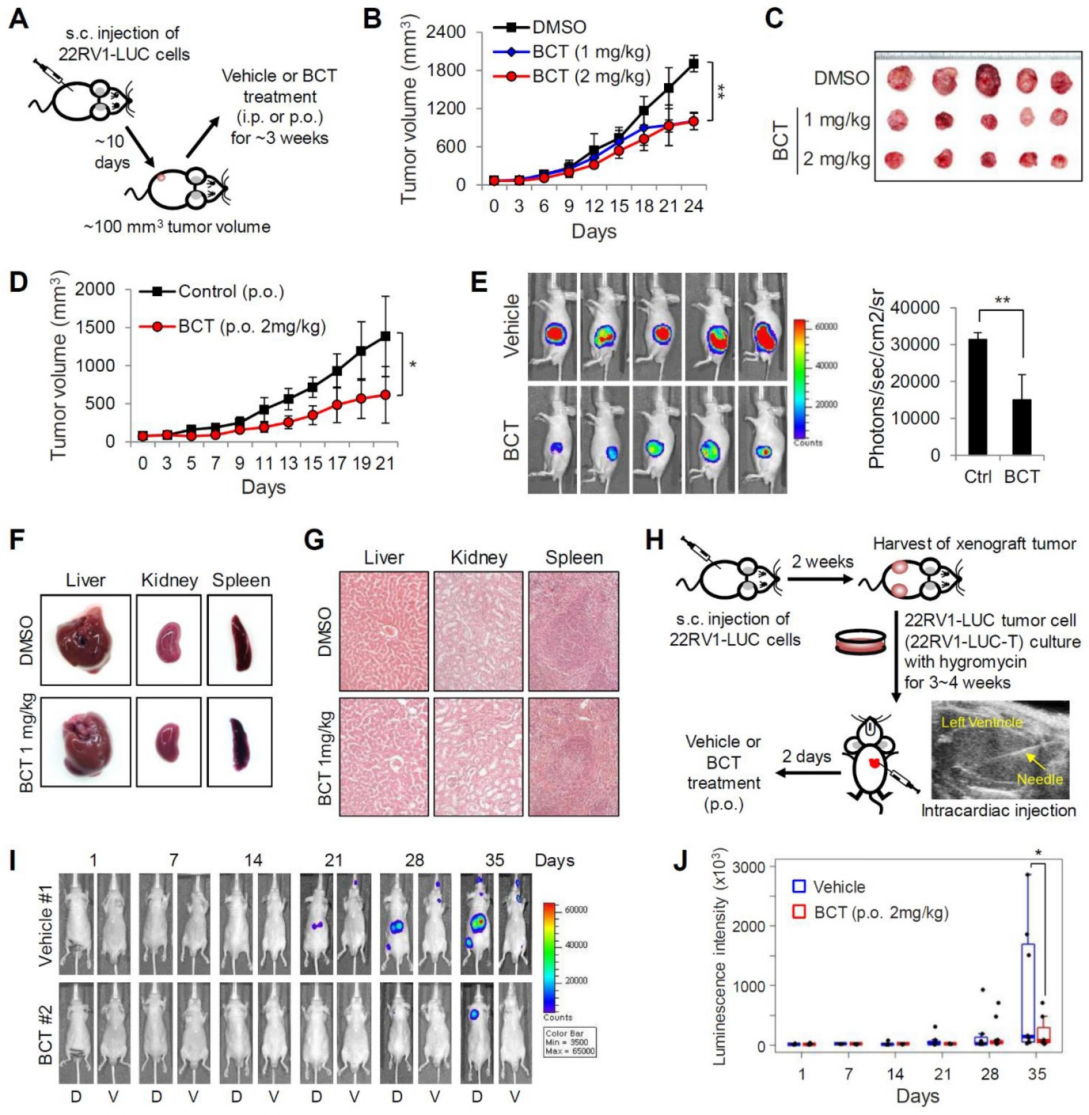
** Inhibitory effects of BCT on the growth and metastasis of CRPC cells in vivo.** (A) Schematic representation of experimental design of s.c. 22RV1-LUC xenograft model and BCT treatment. (B, C) Mice bearing 22RV1-LUC xenografts were treated i.p. with DMSO or BCT once every 3 days (n = 5). Tumor growth curves (B) and extracted tumors (C) are shown. **p < 0.01. (D) Mice bearing 22RV1-LUC xenografts were treated with vehicle or BCT by oral gavage every other day (n = 5). *p < 0.05. (E) Bioluminescence images of tumor-bearing mice are shown (left panel), and the average signal intensity (n = 5) of regions of interest is quantitated (right panel). **p < 0.01. (F, G) Representative liver, kidney, and spleen from the same experiment as (B) were anatomically (F) and histologically (G, H&E staining) evaluated. (H) Schematic diagram of metastasis model of 22RV1-LUC-T cells with intracardiac injection. (I, J) 22RV1-LUC-T cells were intracardially injected in male nude mice, and the mice were treated with vehicle or BCT by oral gavage every other day (n = 8). Representative bioluminescence images of the mice (dorsal and ventral sides) treated with vehicle or BCT for the indicated days of treatment (I) and graphical representation of normalized bioluminescence signals of metastatic tumor development (J) are shown. The boxes represent the 25th-75th percentile range. Wilcoxon two-tailed rank-sum test at day 35, *p < 0.05.

We next examined the in vivo efficacy of BCT on the growth of CRPC xenograft tumors in male BALB/c nude mice injected with 22RV1 cells constitutively expressing luciferase (22RV1-LUC) (Figure 3A). Intraperitoneal administration (i.p.) of BCT (1 mg/kg or 2 mg/kg, once every three days) significantly inhibited the growth of subcutaneously implanted 22RV1 xenograft tumor (Figure 3B-C). Oral administration (p.o.) of BCT (2 mg/kg, once every two days) was also highly effective in inhibiting CRPC tumor growth (Figure 3D-E). In contrast, the CRPC xenografts were resistant to ENZ, despite high dose and frequent administration (10 mg/kg/day) (Figure S4). In addition, BCT treatment (i.p. and p.o.) inhibited the expression of AR-FL/AR-V7 target genes in xenograft tumors (Figure S5). These results suggest that BCT can block ENZ-resistant CRPC tumor growth and in vivo expression of AR-FL/AR-V7 target genes. Both i.p. and p.o. administration of BCT did not show significant differences in the levels of serum alanine transaminase, aspartate transaminase, lactate dehydrogenase, creatinine, and creatine phosphokinase between the treatment and control groups (Figure S6A). In addition, BCT (i.p. and p.o.) neither significantly affected the body weight of mice (Figure S6B-C) nor showed apparent gross anatomical and histological changes in liver, spleen, and kidney tissues (Figure 3F-G and Figure S6D-E), suggesting that BCT inhibits in vivo CRPC tumor growth without apparent cytotoxicity.

Given our results showing inhibitory effects of BCT on in vitro metastatic properties of CRPC cells (Figure 1F, H-I), we evaluated the effect of BCT on metastatic potential of CRPC cells in mice. Because subcutaneous tumor cells did not show metastasis to distant sites (Figure 3E), we utilized a previously established metastasis model involving in vivo enrichment, selection, and intracardial injection of CRPC cells (Figure 3H). After injection of 22RV1-LUC-T cells into the left cardiac ventricle of nude mice, mice were treated orally with vehicle or BCT for 5 weeks (2 mg/kg, once every two days), and the metastatic tumor growth and number of metastases were monitored every week by bioluminescence imaging. As shown in Figure 3I-J and Figure S7A-B, the metastatic growth of CRPC cells were significantly reduced in BCT-treated group compared to those in the control group. BCT treatment also induced 72% reduction in bioluminescence signals of metastatic tumors at the end of treatment (Figure S7C). These results demonstrate that BCT inhibits metastatic spread and growth of CRPC cells.

### BCT promotes degradation of AR-FL/AR-V7 through the ubiquitin-proteasome pathway

**Figure 4:**
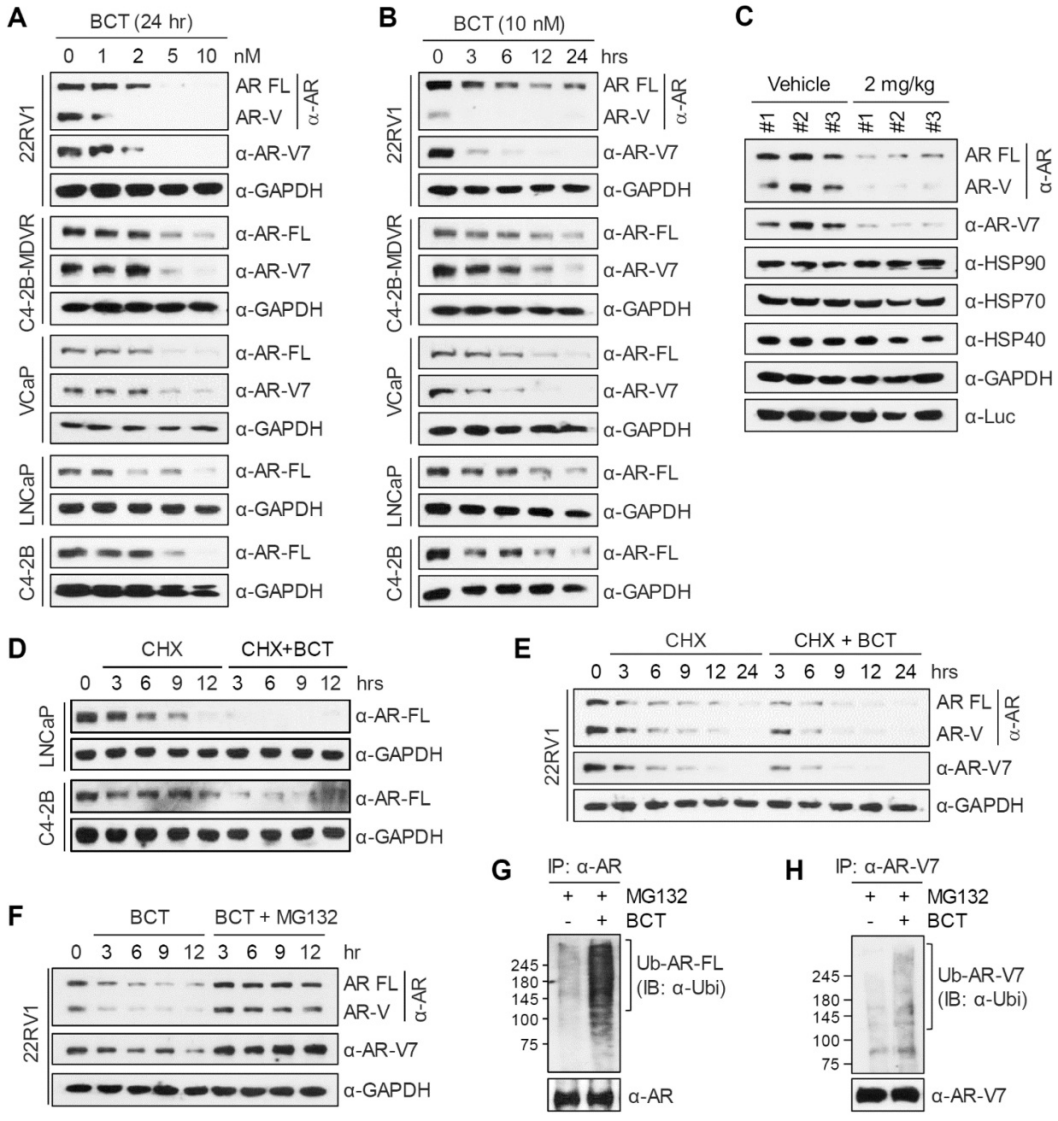
** BCT downregulates AR-FL/AR-V7 levels through the ubiquitin-proteasome pathway.** (A, B) Prostate cancer cells were treated with indicated concentrations (A) and time points (B) of BCT, and cell lysates were analyzed by immunoblot with indicated antibodies. (C) Immunoblot analyses of AR-FL, AR-V7, and HSPs in 22RV1 xenograft tumors treated p.o. with vehicle or BCT. (D, E) LNCaP, C4-2B (D), and 22RV1 (E) cells were treated with cycloheximide (CHX) and BCT as indicated for indicated time periods, and immunoblots were performed with indicated antibodies. (F) 22RV1 cells were treated with 10 nM BCT and 10 μM MG132 as indicated for indicated time periods, and immunoblots were performed with indicated antibodies. (G, H) 22RV1 cell lysates treated with BCT and MG132 as indicated for 4 h were immunoprecipitated with anti-AR (G) or anti-AR-V7 (H) antibody and immunoblotted with indicated antibodies.

To obtain mechanistic insights, we first tested the effect of BCT on AR-FL and AR-V7 protein levels. BCT decreased protein levels of AR-FL and AR-V7 in 22RV1, C4-2B-MDVR, and VCaP cells in a dose and time-dependent manner (Figure 4A-B), without affecting their mRNA levels in 22RV1 cells (Figure 2F). Similar effects of BCT on AR-FL protein levels were observed in LNCaP and C4-2B cells (Figure 4A-B) and in 293T cells ectopically expressing AR-FL under the control of CMV promoter (Figure S8A). BCT also reduced androgen-stabilized AR-FL protein levels in 22RV1 and LNCaP cells (Figure S8B). In addition, AR-FL and AR-V7 protein levels were reduced in BCT-treated xenograft tumors (Figure 4C and Figure S6F). When protein synthesis was blocked by cycloheximide, AR-FL protein levels decreased more rapidly in LNCaP and C4-2B cells in the presence of BCT (Figure 4D), and, similarly, AR-FL and AR-V7 in 22RV1 cells were also rapidly downregulated by BCT (Figure 4E). In addition, MG132, a proteasome inhibitor, reversed BCT-induced degradation of AR-FL in LNCaP and C4-2B cells (Figure S8C) and of AR-FL and AR-V7 in 22RV1 cells (Figure 4F). Importantly, BCT increased the ubiquitination levels of endogenous and ectopically expressed AR-FL and AR-V7 in 22RV1 cells (Figure 4G-H and Figure S8D-E). These results suggest that BCT downregulates AR-FL/AR-V7 expression at the post-translational level and targets these nuclear receptors for degradation through the ubiquitin-proteasome pathway.

### BCT inhibits AR-FL/AR-V7-chaperone complex formation

**Figure 5:**
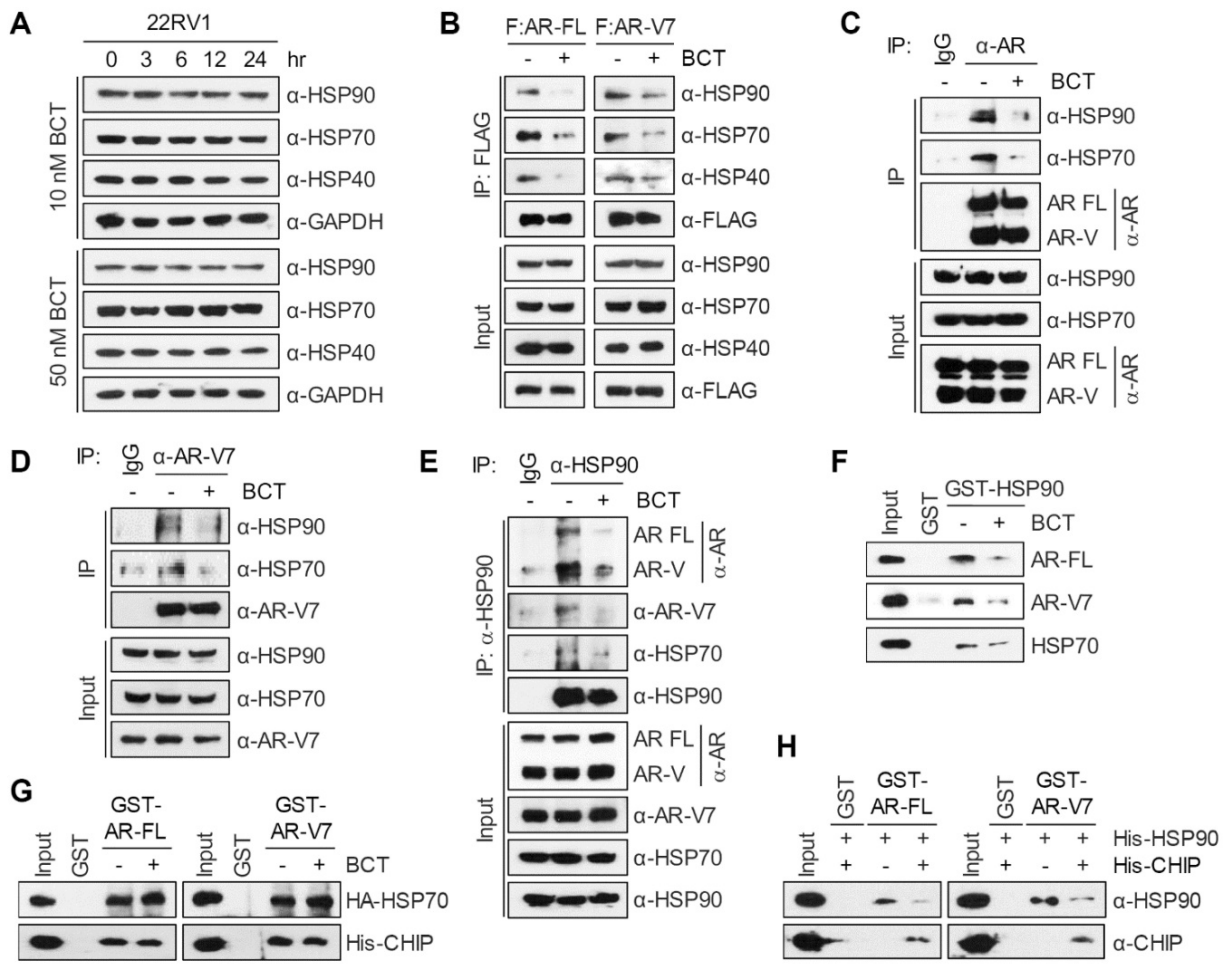
** BCT disrupts the interaction of AR-FL/AR-V7 with molecular chaperones.** (A) 22RV1 cells were treated with BCT for indicated time periods, and cell lysates were analyzed by immunoblot with the indicated antibodies. (B) 22RV1 cells transfected with 3xFLAG-AR-FL or 3xFLAG-AR-V7 expression vector were treated with DMSO or 10 nM BCT for 4 h. Cell lysates were immunoprecipitated with anti-FLAG M2 agarose and immunoblotted with indicated antibodies. (C-E) 22RV1 cell lysates treated with 10 μM MG132 and DMSO or BCT were immunoprecipitated with anti-AR (C), anti-AR-V7 (D), or anti-HSP90 (E) antibody and immunoblotted with indicated antibodies. (F) In vitro-translated HA-AR-FL, HA-AR-V7, and HA-HSP70 were incubated with GST or GST-HSP90 in the presence or absence of BCT. Bound proteins were analyzed by immunoblot with anti-HA antibody. (G) GST pull-down assays were performed using in vitro-translated HA-HSP70 or His-CHIP and indicated GST proteins. Bound proteins were analyzed by immunoblot with anti-HA or anti-His antibody. (H) GST pull-down assays were performed as described in (G), followed by immunoblot with the indicated antibody.

AR molecular chaperones, including HSP90, HSP70, and HSP40, play important roles in stabilizing both unliganded and hormone-bound AR and confer resistance to ENZ 12, 13, 22, 23. Given that BCT downregulates AR-FL and AR-V7 protein levels, we examined the effect of BCT on protein levels of AR molecular chaperones. Unlike the case of AR-FL and AR-V7, BCT had no effect on protein levels of HSP90, HSP70, and HSP40 in 22RV1, C4-2B-MDVR, VCaP, and LNCaP cells (Figure 5A and Figure S9A). We further confirmed these results in the BCT-treated 22RV1 xenograft tumors (Figure 4C and Figure S6F). We next tested whether BCT can affect the interaction of AR-FL/AR-V7 with molecular chaperones. Coimmunoprecipitation (CoIP) experiments showed that BCT inhibits the interaction of ectopically expressed AR-FL and AR-V7 with endogenous HSP90, HSP70, and HSP40 in 22RV1 cells (Figure 5B) and also inhibits interactions between overexpressed AR-FL/AR-V7 and HSPs in 293T cells (Figure S9B-C). Endogenous CoIP experiments confirmed the inhibitory effects of BCT on the interaction of AR-FL and AR-V7 with HSP90 and HSP70 (Figure 5C-D), and the same results were observed in reciprocal endogenous CoIP experiments (Figure 5E) and semi-endogenous CoIP experiments (Figure S9D). These results, together with the results shown in Figure 4, suggest that BCT might induce the degradation of AR-FL/AR-V7 by disrupting their interaction with molecular chaperones. In addition, GST pull-down assays showed that HSP90 and HSP70 interact directly with AR-FL and AR-V7 and that BCT inhibits interactions of AR-FL and AR-V7 with HSP90, but not with HSP70 (Figure 5F-G). Interestingly, BCT also inhibited the interaction between HSP90 and HSP70 (Figure 5E-F and Figure S9D), indicating that BCT targets the interaction of HSP90 with HSP70 as well as with its client proteins AR-FL and AR-V7.

We previously reported that CHIP binds to AR-V7 and promotes ubiquitination-dependent degradation of AR-V7 by acting as an E3 ligase 20. We next examined whether HSP90 and CHIP compete for binding to AR-FL and AR-V7 and found that HSP90 binding to AR-FL and AR-V7 was blocked by CHIP (Figure 5H). In addition, in contrast to the inhibitory effect on HSP90 binding to AR-FL and AR-V7, BCT did not affect the interaction of CHIP with AR-FL and AR-V7 (Figure 5G) but instead increased CHIP-mediated ubiquitination of AR-FL and AR-V7 (Figure S9E-F). Collectively, these results suggest that HSP90 and CHIP compete for binding to AR-FL/AR-V7 and that BCT facilitates CHIP-mediated ubiquitination of AR-FL/AR-V7 by blocking the interaction of HSP90 with AR-FL/AR-V7.

### BCT directly binds to and inhibits the chaperone function of HSP90 in CRPC cells

**Figure 6:**
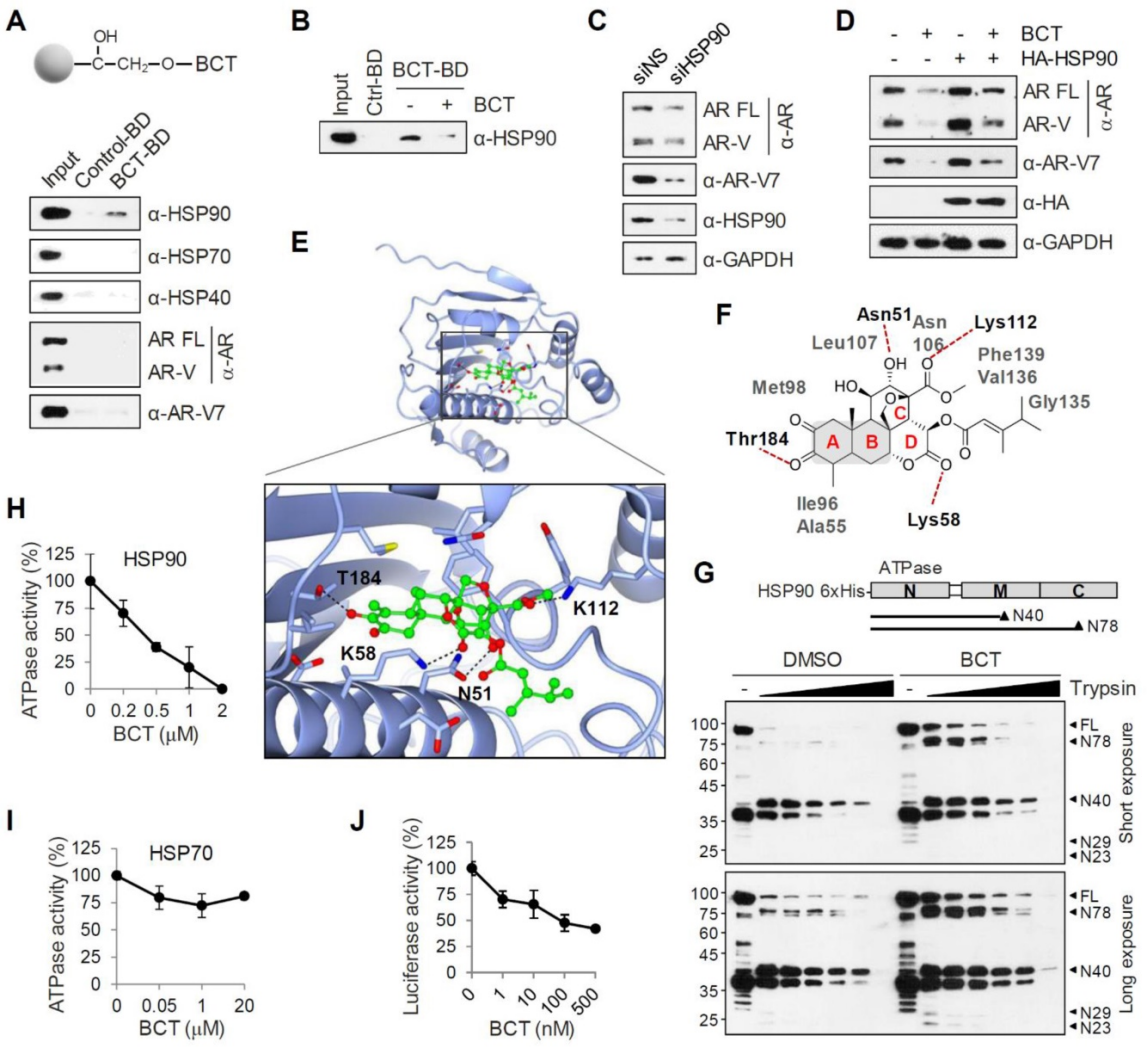
** BCT binds to and interferes with the chaperone function of HSP90.** (A) 22RV1 whole cell extracts were incubated with control or BCT-immobilized beads (upper), and bound proteins were analyzed by immunoblot with indicated antibodies (lower). (B) 22RV1 whole cell extracts were pretreated with DMSO or excess amount of unlabeled BCT (25 μM) for 1 h, followed by pull-down with BCT-immobilized beads and immunoblots as described above. (C) Immunoblot of indicated proteins following RNAi-mediated depletion of HSP90 in 22RV1 cells. (D) 22RV1 cells transfected with empty or HA-HSP90 expression vector were treated with DMSO or 10 nM BCT. Cell lysates were analyzed by immunoblot with the indicated antibodies. (E) Molecular docking model of BCT bound to HSP90. Ribbon view of the HSP90-BCT binding pocket. Amino acid residues involved in BCT binding in HSP90 are labelled. Hydrogen bonds are depicted as dashed lines. (F) Two-dimensional representation of interactions between HSP90 and BCT. (G) Schematic diagram of domain structure of HSP90 (upper). Arrowheads indicate major trypsin cleavage sites. Limited proteolytic digestion of N-terminally His-tagged HSP90 by trypsin (lower). Trypsin-digested fragments were detected by immunoblot with anti-His antibody. (H, I) ATPase activities were measured after incubation of recombinant HSP90 (H) or HSP70 (I) with indicated concentrations of BCT. Data are means ± s.d. (n = 3). (J) Inhibition of luciferase refolding in 22RV1 cells treated with BCT. Luciferase activity is expressed as a percentage of DMSO control. Data are means ± s.d. (n = 3).

To identify BCT-binding proteins among the components of AR-chaperone complexes, we immobilized BCT onto epoxide-activated UNOsphere beads and performed pull-down assays using the BCT affinity resin and 22RV1 whole cell lysates. As shown in Figure 6A, HSP90, but not HSP70, HSP40, AR-FL, and AR-Vs, was captured from 22RV1 lysates with the BCT-conjugated beads. Similarly, BCT-conjugated beads captured ectopically expressed HSP90, but not other HSPs, from 293T cell lysates (Figure S10A). In addition, pretreatment of 22RV1 cell lysates with excess free BCT as a competitor efficiently inhibited HSP90 pull-down by BCT-conjugated beads (Figure 6B), indicating that BCT binds specifically to HSP90. Furthermore, HSP90 silencing decreased AR-FL and AR-V7 protein levels (Figure 6C), and conversely, HSP90 overexpression increased AR-FL and AR-V7 levels and partially reversed BCT-mediated degradation of AR-FL and AR-V7 (Figure 6D). These results further confirm target specificity of BCT and suggest direct involvement of HSP90 in the regulation of AR-FL/AR-V7 protein stability.

HSP90 binds and hydrolyzes ATP using its NTD, and its ATPase activity is essential for client binding and its chaperone activity 15, 16. To gain a better understanding of the binding mode of BCT to HSP90, we performed a molecular docking simulation and identified a potential binding site of BCT in the ATPase domain of human HSP90. BCT fits the ATP binding pocket of HSP90 in a similar fashion as ATP (Figure S10B). The docked conformation of BCT is a good match for that of ATP bound to the pocket (Figure S10C). BCT occupied the binding pocket through hydrogen bonds and various hydrophobic interactions with active site residues (Figure 6E-F). We predicted that the carbonyl oxygens of ring D and methyl carboxylate of ring C form hydrogen bonds with the side chain amides of Lys58 and Lys112, respectively, at the entrance of the pocket and that the tautomeric oxygen of ring A and the hydroxyl oxygen of ring C also form hydrogen bonds with the side chains of Thr184 and Asn51. The rings A and B of BCT fitted into the bottom of the ATP-binding pocket and form a sandwiched hydrophobic interaction with Ala55, Ile96, Met98, and Leu107 (Figure 6F).

Inhibitor binding often induces conformational changes in HSP90 and facilitates dissociation of client proteins from HSP90, leading to their degradation through the ubiquitination-proteasome pathway 24. To investigate the conformational changes in HSP90 induced by BCT binding, we performed limited proteolytic digestion analysis of N-terminally His-tagged HSP90 with various amounts of trypsin and analyzed the proteolytic fragments of HSP90 by immunoblot with anti-His antibody. The proteolytic pattern of HSP90 was in agreement with previous reports with two predominant cleavage sites in the C-terminal and the middle domain (Figure 6G). Interestingly, even though BCT-bound HSP90 showed a similar proteolytic profile to DMSO control, BCT-bound HSP90 FL and its N-terminal fragments including N78, N40, N29, and N23 were greater resistant to trypsin digestion. Because BCT confers tryptic resistance not only to HSP90 FL but also to its N-terminal fragments, these results suggest that BCT induces conformational changes of HSP90 by binding to the NTD of HSP90.

We next evaluated the effects of BCT on the ATPase activity of HSP90 and found that BCT inhibited the ATPase activity of HSP90 in a dose-dependent manner (Figure 6H). In addition, consistent with the results showing specific binding of BCT to HSP90 but not to HSP70 (Figure 6A), BCT did not significantly affect the ATPase activity of HSP70 even at higher concentration than that used for complete inhibition of HSP90 activity (Figure 6I). To further investigate whether BCT can inhibit HSP90 chaperone function, we performed cell-based luciferase reactivation assay, which employs luciferase as the reporter enzyme to be refolded by the HSP90-dependent refolding system. 22RV1 cells were treated with cycloheximide to block de novo luciferase synthesis, heat denatured, and then incubated at 37ºC to allow for luciferase refolding. Luciferase activity under these conditions increased 5-fold from denatured levels, and BCT prevented the HSP90-dependent refolding of luciferase at nanomolar concentrations (Figure 6J). These results suggest that BCT inhibits ATPase-dependent chaperone function of HSP90.

### HSP90 expression is upregulated and correlates with AR expression in PCa tumors

**Figure 7:**
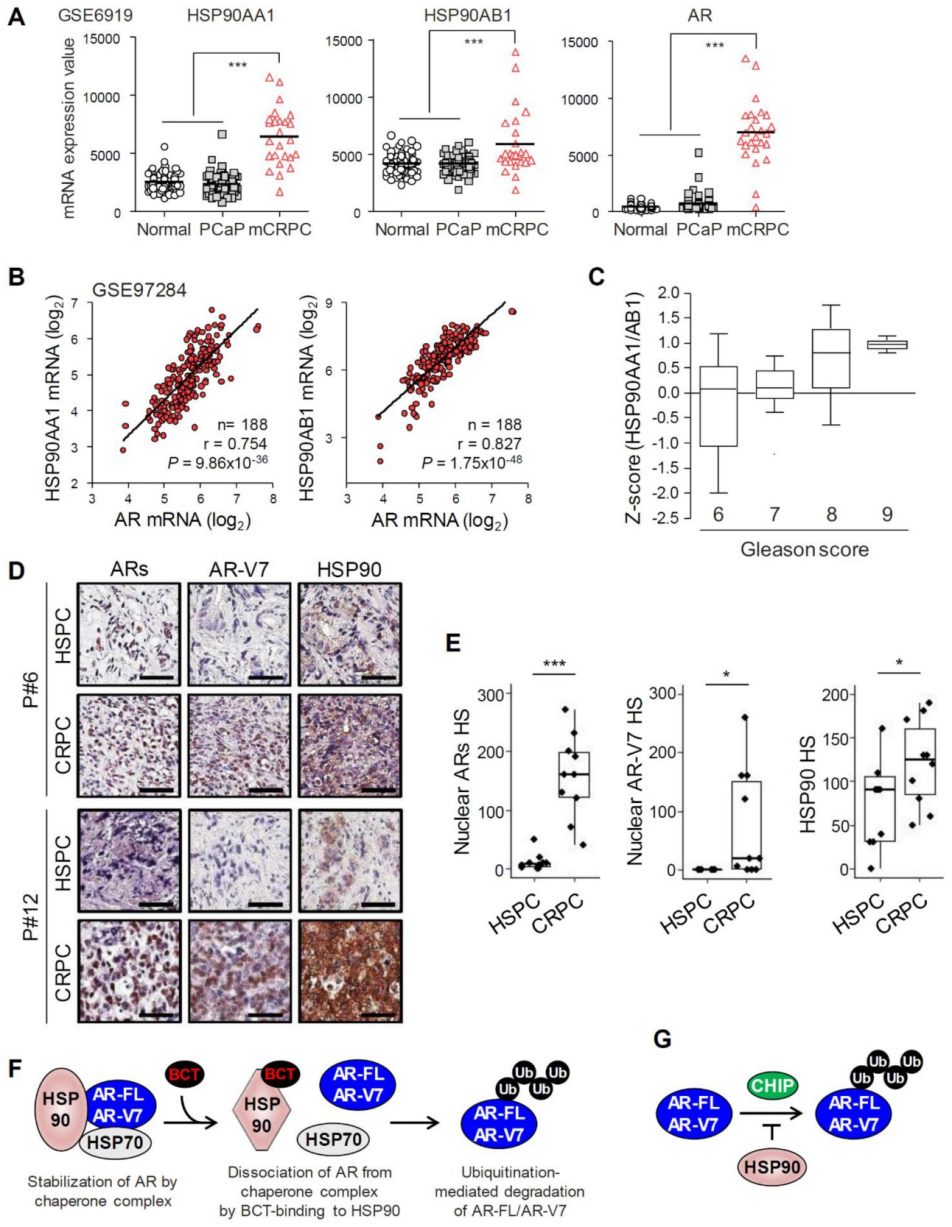
** HSP90 expression is correlated with PCa progression and with levels of AR/AR-V7.** (A) HSP90 gene (HSP90AA1 and HSP90AB1) and AR expression levels were determined in normal, primary PCa (PCaP), and metastatic CRPC (mCRPC). Statistical analysis was performed using one-way ANOVA. ***p < 0.001. (B) Correlation analysis of HSP90 and AR expression in PCa (GSE97284). (C) The association between HSP90AA1/AB1 expression and Gleason score was evaluated in Singh Prostate dataset. (D) Representative immunohistochemistry images of ARs, AR-V7, and HSP90 in patient-matched HSPC and CRPC tissues (patient #6 and #12). (E) Nuclear ARs and AR-V7 and total HSP90 H-score (HS) for matched HSPC and CRPC tissues. ***p < 0.001, *p < 0.05. (F) Working model of BCT-mediated degradation of AR-FL/AR-V7 in CRPC cells. BCT binds to HSP90 and inhibits AR-HSP90 chaperone complex formation by inducing a conformational change of HSP90, resulting in CHIP-mediated ubiquitination and degradation of AR-FL/AR-V7. (G) The role of HSP90 in the regulation of AR-FL/AR-V7 ubiquitination. HSP90 inhibits ubiquitination of AR-FL/AR-V7 by blocking CHIP binding to AR-FL/AR-V7.

We analyzed the expression levels of HSP90 in normal prostate and PCa tissues using Oncomine and GEO databases. HSP90 isoforms (HSP90AA1 and HSP90AB1) and AR-FL mRNA levels were increased in PCa tissues compared to normal prostate tissues in three independent Oncomine microarray datasets (Figure S11). In addition, mRNA levels of HSP90 isoforms and AR-FL were significantly upregulated in metastatic CRPC compared to normal prostate or benign prostatic hyperplasia and primary PCa in two independent GEO microarray datasets (Figure 7A and Figure S12A), and AR-FL expression was significantly correlated with levels of HSP90 isoforms (Figure 7B). Furthermore, HSP90 expression level increased with increasing Gleason score (Figure 7C). Together, these results suggest that HSP90 and AR-FL are upregulated in advanced CRPC and that their expression is correlated with each other and with PCa progression.

Recently, we reported that AR-FL and AR-V7 mRNA levels are significantly upregulated in CRPC compared to patient-matched primary PCa 25. We further evaluated expression levels of ARs (AR-FL and AR-Vs), AR-V7, and HSP90 at the histological level in matched samples of primary hormone-sensitive PCa (HSPC) and CRPC from ten metastatic CRPC patients who had progressed on androgen deprivation therapy. Nuclear ARs and AR-V7 levels significantly increased in 100% (10/10) and 70% (7/10), respectively, of CRPC tissues examined compared to matched HSPC tissues, and HSP90 expression also increased in 80% (8/10) of CRPC tissues (Figure 7D-E), suggesting their role in progression to CRPC. Moreover, among eight CRPC tissues with increased HSP90 expression, eight cases (100%) had increased levels of ARs, and five cases (62.5%) had increased AR-V7 expression (Figure S12B), suggesting a correlation between the expression levels of HSP90 and ARs/AR-V7 in CRPC.

## Discussion

Current therapies for PCa have focused on blocking androgen-dependent activation of AR-FL and reducing de novo androgen production 1, 4. However, PCa eventually escapes its response to hormone therapies and progresses to ENZ/ABI-resistant CRPC by developing multiple mechanisms including expression of constitutively active AR-Vs lacking the LBD, especially AR-V7 1-5. Thus, identification of novel drugs simultaneously targeting both AR-FL and AR-V7 activities would help overcome these clinical challenges and provide novel therapeutic options in the treatment of HSPC and CRPC. In this study, we have identified BCT as a potent inhibitor of AR-FL and AR-V7 signaling in CRPC cells. BCT blocked the transcriptional activities of both ligand-dependent AR-FL and constitutively active AR-V7 and also inhibited CRPC cell growth by downregulating AR-FL/AR-V7 protein levels and their transcriptional programs. Furthermore, BCT exhibited potent anti-tumor and anti-metastatic activity against CRPC xenografts in mice.

BCT is a natural tetracyclic triterpene quassinoid isolated from Brucea species and has been reported to have anticancer activity against cancer cells including leukemia, lymphoma, and myeloma cells 26, 27, as well as antimalarial activity 28. However, its mechanism of action and molecular targets in cancer cells have not been fully explored. Here, we explored the mechanism of BCT-induced AR-FL/AR-V7 degradation and direct cellular target of BCT and found that BCT blocks AR-FL/AR-V7-chaperone complex formation, consequently leading to degradation of AR-FL/AR-V7 through ubiquitin-proteasome pathway (Figure 7F). BCT could bind directly to the ATP-binding pocket of HSP90, induce a conformational change, and inhibit ATPase and chaperone activity of HSP90, indicating that BCT is an ATP competitive inhibitor of HSP90. Thus, our findings suggest BCT as a novel HSP90 inhibitor with potential for the treatment of CRPC patients with AR-FL/AR-V7-positive tumors and provide rationale targeting HSP90 as a potential therapeutic strategy to overcome resistance to AR LBD-targeted hormone therapies in CRPC.

Drug repositioning has gained considerable attention as an effective strategy to find new targets and indications for existing drugs and compounds 29. Chloroquine, metformin, artemisinin, dihydroartemisinin, artesunate, ferroquine, and quinacrine, which were originally used as antimalarials, have been tested for their anticancer activity and proved to be potent anticancer agents against PCa and CRPC cells 18, 19, 30-34. In addition, a recent paper showed that the antimalarial quassinoid AIL, which was also identified in our screen, can inhibit the growth and metastasis of PCa cells in mouse CRPC models 17. Interestingly, anticancer HSP90 inhibitors have been reported to have considerable therapeutic potential for malaria 35. These previous results, together with our findings showing potent anti-CRPC activity of BCT, demonstrate the potential of repurposing a certain class of antimalarials for CRPC treatment and of HSP90 as a common therapeutic target for cancer and malaria.

We previously reported that CHIP, a co-chaperone and E3 ubiquitin ligase, interacts with and downregulates AR-V7 levels by ubiquitinating and targeting AR-V7 for proteasomal degradation 20. A recent paper also showed that CHIP blocks complex formation between AR-FL/AR-V7 and HSP70, leading to AR-FL/AR-V7 ubiquitination and degradation 22. However, the regulation mechanism of AR-V7 activity and stability by molecular chaperones still remains largely unknown. In this study, we provided several lines of evidence that AR-V7 is a bona fide client of HSP90 in CRPC cells: HSP90 binds AR-V7 as well as AR-FL and competes with CHIP for binding to them; HSP90 knockdown decreases AR-FL/AR-Vs protein levels; and HSP90 overexpression stabilizes AR-FL/AR-Vs levels and partially blocks their degradation by BCT-mediated HSP90 inhibition. Thus, HSP90 contributes to CRPC progression, probably not only through its chaperone activity for AR-FL/AR-V7 (Figure 7F) but also by blocking CHIP-mediated AR-FL/AR-V7 ubiquitination and degradation (Figure 7G). Consistent with our results, a recent study using CoIP combined with biomass spectrum assay also showed that AR-V7 can associate with HSP90 in 22RV1 cells 36. However, it should be noted that previous studies showed that HSP90 interacts with AR-FL but not with AR-V7 lacking the LBD 37, 38. Thus, we cannot rule out the possibility that HSP90 inhibition by BCT affects AR-V7 stability indirectly through downregulating AR-V7 coregulators which protect AR-V7 from ubiquitin-mediated degradation. Furthermore, HSP90 levels are significantly upregulated in metastatic CRPC compared to normal prostate and primary PCa and correlates with Gleason score and AR-FL/AR-V7 expression. Therefore, our results demonstrate the involvement of HSP90 in CRPC progression as well as in the regulation of AR-FL/AR-V7 and suggest that targeting HSP90 might be a promising selective therapeutic strategy to treat CRPC patients with high AR-FL/AR-V7 and HSP90 expression.

HSP90 is a molecular chaperone that facilitates the conformational maturation of more than 200 proteins including cell survival and oncogenic signaling proteins, such as MYC, AKT, and nuclear receptors including AR and glucocorticoid receptor (GR) 13, 16, which play important roles in the development and progression of PCa. Thus, HSP90 inhibition may affect the stability of other oncogenic HSP90 clients in addition to AR-FL/AR-V7 in cancer cells. Consistent with this notion, HSP90 inhibitors 17-AAG and PU-H71 have been shown to reduce MYC and AKT protein levels and expression of their target genes in cancer cells 39. Importantly, we also found that BCT repressed the MYC transcriptional program (Figure 2C) and reduced MYC, AKT, and GR protein levels in CRPC cells (Figure S13). Interestingly, AKT gene expression is preferentially upregulated by AR-V7 but not by AR-FL 40, and AR-V7 activity can be inhibited by AKT inhibitors 41, suggesting a positive feedback loop between AKT expression and AR-V7 activation in CRPC cells. In addition, previous work revealed that AR and GR share largely overlapping cistromes and transcriptomes in CRPC cells and that increased GR expression, a common feature of ENZ resistance, enhances AR target gene expression required for CRPC cell growth, indicating an interplay between nuclear receptors in CRPC 42. Thus, BCT-mediated inhibition of HSP90 chaperone function might provide a multifaceted approach to targeting heterogeneous metastatic CRPCs not only by promoting degradation of various oncogenic proteins in CRPC cells but also by inhibiting interplays and feedback loops of oncogenic signaling pathways.

HSP90 has been extensively investigated as a promising anticancer target 13. However, although HSP90 inhibitors showed promising activity in preclinical studies on various cancers including PCa, the use of HSP90 inhibitors has not been successful in clinical studies. Recently, ganetespib and onalespib, second generation HSP90 inhibitors, have been reported to have minimal clinical activity in patients with metastatic CRPC 43, 44. Most of HSP90 inhibitors targeting the NTD of HSP90 induce cytoprotective heat shock response mediated by HSF1, which results in the increased expression of inducible HSPs (*e.g.*, HSP70, HSP40, and HSP27) 13. This compensatory mechanism causes anti-apoptosis and drug resistance and limits the clinical impact of HSP90 inhibitors. Thus, development of HSP90 inhibitors that act without stimulating heat shock response could provide substantial benefits to patients with CRPC and other types of cancer. Importantly, BCT reduced 22RV1 cell viability and protein levels of AR-FL and AR-V7 more potently than the first (17-AAG) and second (onalespib and AUY-922) generation HSP90 inhibitors but showed no effect on the expression of HSP70 and HSP40, which is activated *via* the heat shock response induced by previous generations of HSP90 inhibitors (Figure S6G-I), indicating that BCT does not induce cellular heat shock response in CRPC cells and also suggesting that BCT may have an advantage over other HSP90 inhibitors in the treatment of CRPC.

In conclusion, we identified and characterized BCT as a promising therapeutic candidate against CRPC. Our preclinical data showed that BCT is efficacious in inhibiting growth and metastasis of CRPC cells through targeting HSP90. Our findings provide a proof-of-concept that targeting HSP90 may be a promising strategy to treat CRPC that has become resistant to conventional hormone therapies.

## Supplementary Material

Supplementary materials and methods, figures, tables.

## Abbreviations

AR: androgen receptor
AR-FL: full-length androgen receptor
AR-V7: androgen receptor variant 7
AR-Vs: androgen receptor variants
PCa: prostate cancer
CRPC: castration-resistant prostate cancer
BCT: bruceantin
NTD: N-terminal domain
LBD: ligand-binding domain
ABI: abiraterone
ENZ: enzalutamide
BIC: bicalutamide
HSP90: heat shock protein 90
BST: brusatol
BCN-A: bruceine A
AIL: ailanthone
DHT: dihydrotestosterone
RNA-seq: RNA-sequencing
GSEA: gene set enrichment analysis
qRT-PCR: Real-time quantitative reverse transcription polymerase chain reaction
i.p.: intraperitoneal administration
p.o.: oral administration
CoIP: coimmunoprecipitation
