# Supplementary material for: Bruceantin targets HSP90 to overcome resistance to hormone therapy in castration-resistant prostate cancer
Source: Theranostics. 2021 Jan 1;11(2):958–73. doi: 10.7150/thno.51478 (PMC7738850; doi:10.7150/thno.51478)
Supplement: Supplementary file 1 — Supplementary materials and methods, figures, tables. [file thnov11p0958s1.pdf]

Supplementary Information

Bruceantin targets HSP90 to overcome resistance to hormone therapy in castration-resistant prostate cancer

This PDF file includes: Supplementary Materials and Methods, Supplementary References, Supplementary Figures S1 to S13, and Supplementary Tables S1 to S4.

Supplementary Materials and Methods

Transient transfection and plasmids. Cells were transiently transfected with MMTV-LUC reporter or expression plasmids for AR-FL, AR-V7, HSP90, HSP70, or HSP40 using Lipofectamine 3000 (ThermoFisher Scientific). HSP90, HSP70, and HSP40 cDNAs were obtained from Addgene (HSP90 HA, #22487) or PCR amplified from 22RV1 cDNA and cloned into pSG5.HA (pSG5.HA-HSP70, pSG5.HA-HSP40), pGEX-4T-1 (pGEX-4T-1-HSP90, pGEX-4T-1-HSP70), or pETDuet-1 (pETDuet-1-HSP90). The following plasmids were described previously[1, 2]: MMTV-LUC, pcDNA3.1-3xFLAG-AR-FL, pcDNA3.1-3xFLAG-AR-V7, pcDNA3.1-3xFLAG-AR-V7-1-360, pcDNA3.1-3xFLAG-AR-V7-361-645, pSG5.HA-AR-FL, pSG5.HA-AR-1-600, pSG5.HA-AR-591-919, pSG5.HA-AR-V7, pSG5.HA-CHIP, pGEX-4T-1-AR-FL, pGEX-4T-1-AR-V7, and pETDuet-1-CHIP.

Cell growth inhibition, colony formation, migration, invasion, and sphere formation assays. PCa cells and normal prostate epithelial cells were seeded in 96-well plates ($1x10^4$ cells/well) and then treated next day with compounds at the indicated concentrations in the figure for 3 days. Cell growth inhibition was determined by MTT assays (Promega). IC50 values were determined by non-linear regression using PRISM v5.0 (GraphPad). For colony formation assays, cells were seeded into 6 well plates ($1x10^3$ cells/well) and treated with compounds at the indicated concentrations in the figure for 14 days. Crystal violet-stained colonies were solubilized in 10% SDS, and absorbance was measured at 570 nm. Two-chamber cell migration and invasion assays were performed in 24-well plates using Transwell inserts (Costar) coated with fibronectin (Sigma-Aldrich) and with Matrigel (BD Biosciences)/fibronectin, respectively. 22RV1 cells ($1x10^5$ cells/well) in serum-free medium were pretreated with 5 μg/ml mitomycin C (Sigma-Aldrich) for 2 h to avoid the effect of BCT on cell proliferation and then loaded into the upper chamber and treated with DMSO or BCT (10 nM or 20 nM). Following 18 hr incubation, migrated or invaded cells in the lower chamber with medium supplemented with 10% FBS as chemoattractant were fixed with methanol and then stained with hematoxylin and eosin (H&E). Images of the cells on the lower layer of the chamber were captured and counted in four random fields. Each assay was performed in triplicate. For sphere formation assays, 22RV1 cells ($2x10^2$ cells/well) were cultured in suspension to form spheres in 96-well ultralow attachment plates (Costar) with serum-free DMEM-F12 (1:1) supplemented with 20 ng/μl hEGF, 10 ng/μl bFGF, 1x B27, and 1x N-2 (Life technologies). After 14 days, the number of spheres with a diameter of >100 μm was counted.

Cell apoptosis analysis. 22RV1 cells were treated with 10 nM BCT for the indicated time in the figure, trypsinized, and washed with phosphate-buffered saline (PBS). Apoptotic cells were stained with propidium iodide (PI) and Annexin V-FITC following the manufacturer's instructions (BD Biosciences) and detected by flow cytometry analysis using a BD FACSVerse flow cytometer (BD Biosciences).

Gene set enrichment analysis (GSEA). GSEA was performed using the Java GSEA software package (http://www.broadinstitute.org/gsea) and run in pre-ranked mode to identify enriched signatures. We used the gene sets in the Molecular Signatures Database (MSigDB) and added Androgen_UP, Androgen_DOWN (genes upregulated and downregulated in response to androgen)[3] and ARV_Activated, ARV_Repressed (genes activated and repressed by AR-Vs)[4]. The GSEA plot, normalized enrichment score (NES), nominal p values (NOM p-val), and false discovery rate q-values (FDR q-val) were derived from GSEA output.

Real-time quantitative reverse transcription PCR (qRT-PCR). Total RNA was extracted using TRIzol Reagent (ThermoFisher Scientific), and qRT–PCR was performed with One-step PrimeScript RT-PCR (Perfect Real Time) Kit (Takara Bio). qRT-PCR was performed in triplicate using gene-specific primers on ABI Prism 7900HT Fast real-time PCR system (Applied Biosystems). Each reaction was normalized to GAPDH or β-actin mRNA levels. The primer sequences used are listed in Table S3.

Histological analysis. Freshly isolated mouse organs and prostate tumors were fixed in 10% neutral-buffered formalin (Sigma-Aldrich), progressively dehydrated in increasing concentrations of ethanol, embedded into paraffin blocks, and sectioned at a thickness of 5 μm. Slide-mounted sections were deparaffinized by xylene, rehydrated in serial dilutions of ethanol, and stained with H&E. For immunohistochemistry, sections of patient-matched PCa tissues (primary HSPC and recurrent CRPC following androgen deprivation therapy) from our previous study[5] were deparaffinized with xylene, rehydrated in serial dilutions of alcohol, subjected to heat-induced antigen retrieval (for 18 min at 800 W microwave; for AR-V7 staining, Tris-EDTA buffer pH8.0; for AR and HSP90 staining, in citrate buffer pH6.0), and blocked with blocking solution (Dako). Sections were incubated with anti-AR-V7 antibody (clone RM7, 1:500), anti-AR antibody (N-20, 1:1,000), or anti-HSP90 antibody (clone F-8, 1:800) for 1 hr. After washes, reactions were visualized using EnVision Detection System (Dako) and DAB chromogen. Sections were counterstained with hematoxylin. Digital images of sections were captured using the Aperio ScanScope XT slide scanner (Aperio Technologies). Cases were scored by

a pathologist (G.Y.K.) blinded to clinical data using the modified H score (HS) method[6]. All procedures involving human subjects were approved by the Institutional Review Board of the Samsung Medical Center.

Immunoblot, coimmunoprecipitation (CoIP), and GST pull-down. For immunoblot, cells were lysed in RIPA buffer (50 mM Tris–HCl, pH 7.5, 150 mM NaCl, 1% NP-40, 0.5% deoxycholate and 0.1% SDS) or FLAG lysis buffer (50mM Tris–HCl, pH 8.0, 137 mM NaCl, 1 mM EDTA, 1% Triton-X 100, 0.2% Sarkosyl, 10% glycerol) with protease inhibitor cocktail (Roche). Whole cell extracts were resolved by SDS-PAGE, transferred to PVDF (Millipore), and probed with specific antibodies as indicated in figures. AR antibody (N-20 or 441) was used to detect both AR-FL and AR-Vs. For CoIP, cell lysates were immunoprecipitated with specific antibodies or control IgG and protein A/G-agarose beads (Santa Cruz Biotechnology), and immunoprecipitates were analyzed by immunoblot as indicated in figures. For GST pull-down assays, purified recombinant or in vitro translated proteins were incubated with either immobilized GST or GST-fusion proteins, and bound proteins were detected with specific antibodies as indicated in figures.

Antibodies. The following antibodies were used in this study: AR (441), AR (N-20), AR (C-19), HSP90 (F-8), HSP70 (H-2), HSP70 (3A3), ubiquitin (P4D1), c-Myc (9E10), GR (M-20), His-probe (H-3), and luciferase (C-12) (Santa Cruz Biotechnology); HSP40 (ab69402) (Abcam); AR-V7 [RM7] (GTX33604) (GeneTex); AR-V7 (AG10008) (Precision Antibody); CHIP (C3B6), AKT2 (D6G4) (Cell Signaling Technology); HA (3F10) (Roche); FLAG M2 and FLAG M2-agarose (Sigma-Aldrich); GAPDH (LF-PA0018) (AbFrontier).

Limited proteolysis assays. Partial proteolytic digests were performed at 30ºC for 15 min by adding trypsin (0.4, 0.8, 1.2, 1.6, 2.0, 2.4 μg/ml) to recombinant N-terminal His-tagged HSP90 protein in PBS. Proteolytic fragments were separated on SDS-PAGE gels, and digested fragments were detected by immunoblot with anti-His antibody.

ATPase activity assays. ATPase assays were performed for 3 hr at 37ºC with recombinant HSP90 or HSP70 protein in reaction buffer (100 mM Tris-HCl, pH7.4, 20 mM KCl, 6 mM MgCl2, and 10 μM ATP) in the presence or absence of BCT (0.05, 0.2, 0.5, 1, 2, 20 μM). ATP depletion and ADP detection were performed using the ADP-Glo Kinase/ATPase assay kit (Promega), and luminescence was measured using the Mithras LB940 multimode plate reader (Berthold).

Cell-based luciferase refolding assays. 22RV1-LUC cells were heat-denatured at 50ºC for 7 min, which was determined as the optimal time and temperature for luciferase denaturation without affecting the viability of cells. Cells were seeded into 96-well plates ($5x10^4$ cells/well), treated with DMSO or BCT (1, 10, 100, 500 nM), and incubated at 37ºC for 1 hr to allow luciferase refolding. Luciferase activity was measured using Firefly Luciferase Assay kit (Promega).

Data availability. All RNA-seq data have been deposited in the GEO database under accession number GSE145790. The hyperlink of the dataset is: https://www.ncbi.nlm.nih.gov/geo/query/acc.cgi?acc=GSE145790. All data are available from the authors upon reasonable request.

Supplementary References

1. Moon, SJ, Jeong, BC, Kim, HJ, Lim, JE, Kwon, GY, and Kim, JH. (2018). DBC1 promotes castration-resistant prostate cancer by positively regulating DNA binding and stability of AR-V7. Oncogene. *37*, 1326-1339.
2. Seo, WY, Jeong, BC, Yu, EJ, Kim, HJ, Kim, SH, Lim, JE, *et al.* (2013). CCAR1 promotes chromatin loading of androgen receptor (AR) transcription complex by stabilizing the association between AR and GATA2. Nucleic Acids Res. *41*, 8526-8536.
3. Mendiratta, P, Mostaghel, E, Guinney, J, Tewari, AK, Porrello, A, Barry, WT, *et al.* (2009). Genomic strategy for targeting therapy in castration-resistant prostate cancer. J Clin Oncol. *27*, 2022-2029.
4. He, Y, Lu, J, Ye, Z, Hao, S, Wang, L, Kohli, M, *et al.* (2018). Androgen receptor splice variants bind to constitutively open chromatin and promote abiraterone-resistant growth of prostate cancer. Nucleic Acids Res. *46*, 1895-1911.
5. Park, HK, Lim, SD, and Kwon, GY. (2019). mRNA expressions of androgen receptor and its variants in matched hormone-sensitive and castration-resistant prostate cancer. Scand J Urol. *53*, 365-371.
6. Detre, S, Saclani Jotti, G, and Dowsett, M. (1995). A "quickscore" method for immunohistochemical semiquantitation: validation for oestrogen receptor in breast carcinomas. J Clin Pathol. *48*, 876-878.

Supplementary Figures and Tables

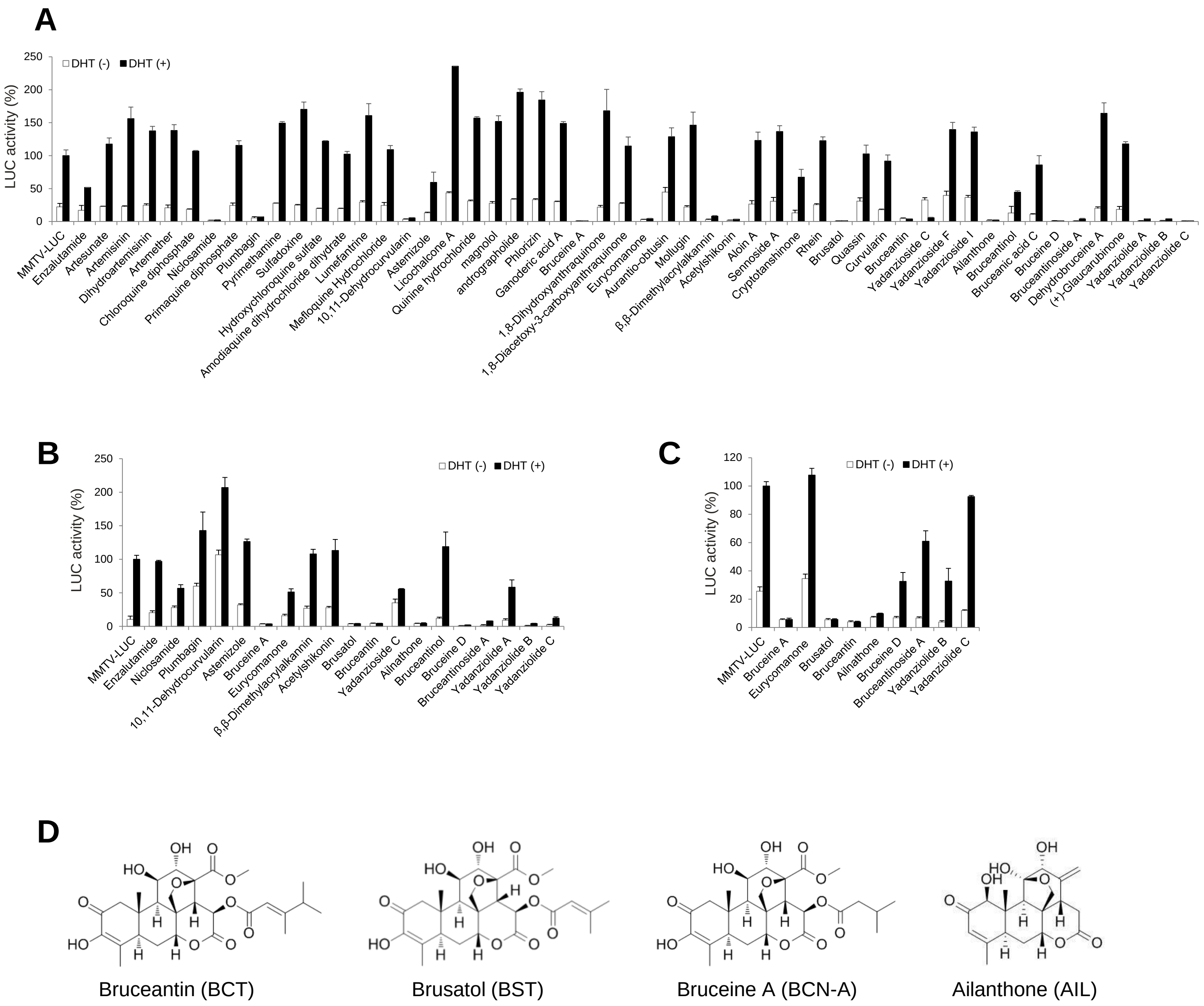

Figure S1. Luciferase assay-based screening of compounds for inhibitory activity against AR-V7 (DHT-) and AR-FL (DHT+) transcriptional activities in 22RV1 cells. (A-C) 22RV1 cells transfected with MMTV-LUC were treated with indicated compounds at 10 μM (A), 1 μM (B), and 0.1 μM (C) with or without 20 nM DHT for 24 hr, and luciferase activities were measured. (D) Identification of four quassinoids as inhibitors of the transcriptional activity of AR-FL and AR-V7.

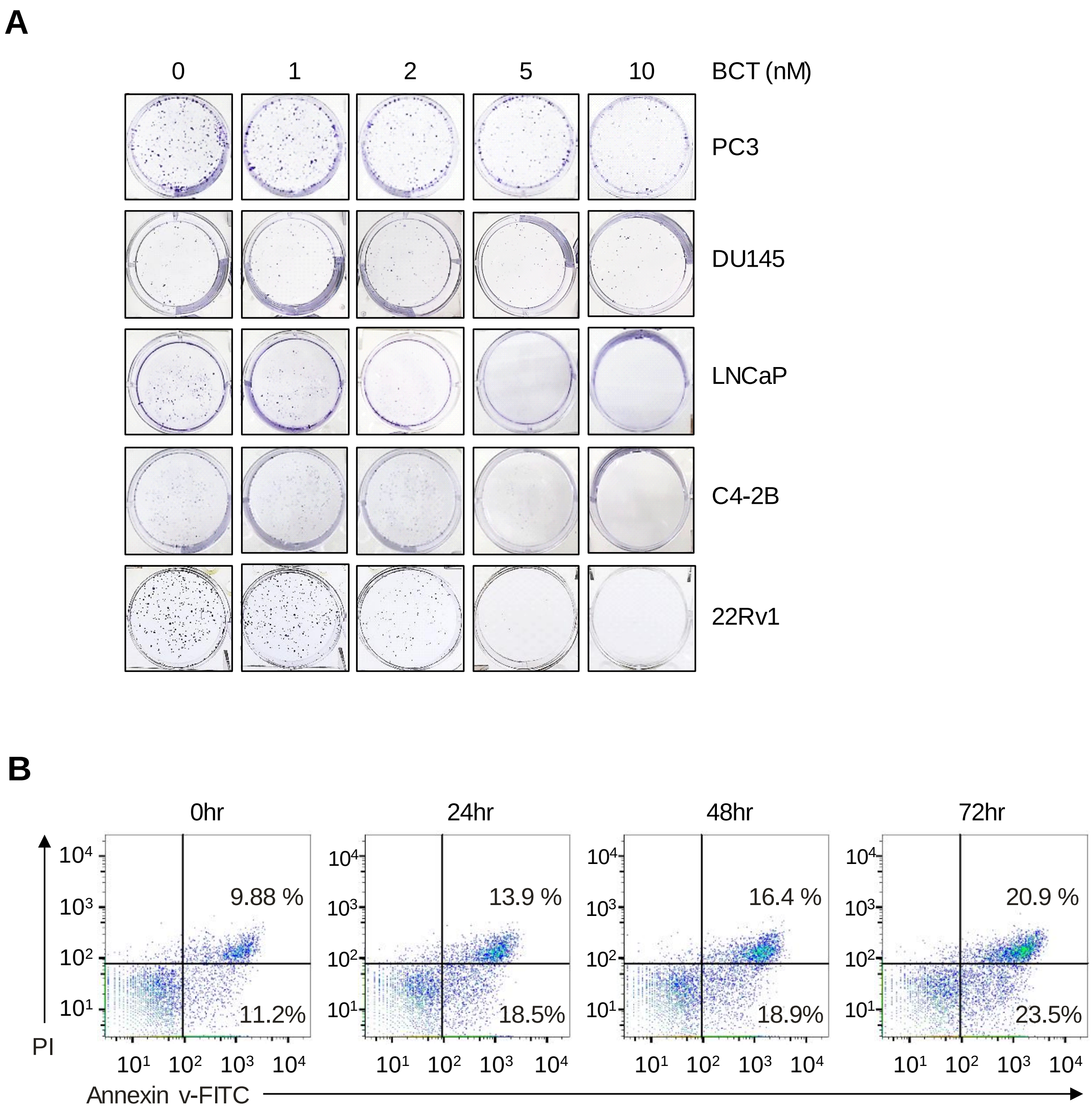

Figure S2. BCT inhibits colony formation and induces apoptosis in 22RV1 cells. (A) Prostate cancer cells were plated and treated with indicated concentrations of BCT for 7 days. Viable colonies were stained with crystal violet, and the dye was extracted and quantified by spectrophotometry (Fig. 1F). (B) BCT induces apoptosis in CRPC cells. 22RV1 cells were treated with 10 nM BCT for the indicated times. To quantify apoptotic cells, cells were stained with propidium iodide (PI) and Annexin V-FITC and subjected to flow cytometry analysis.

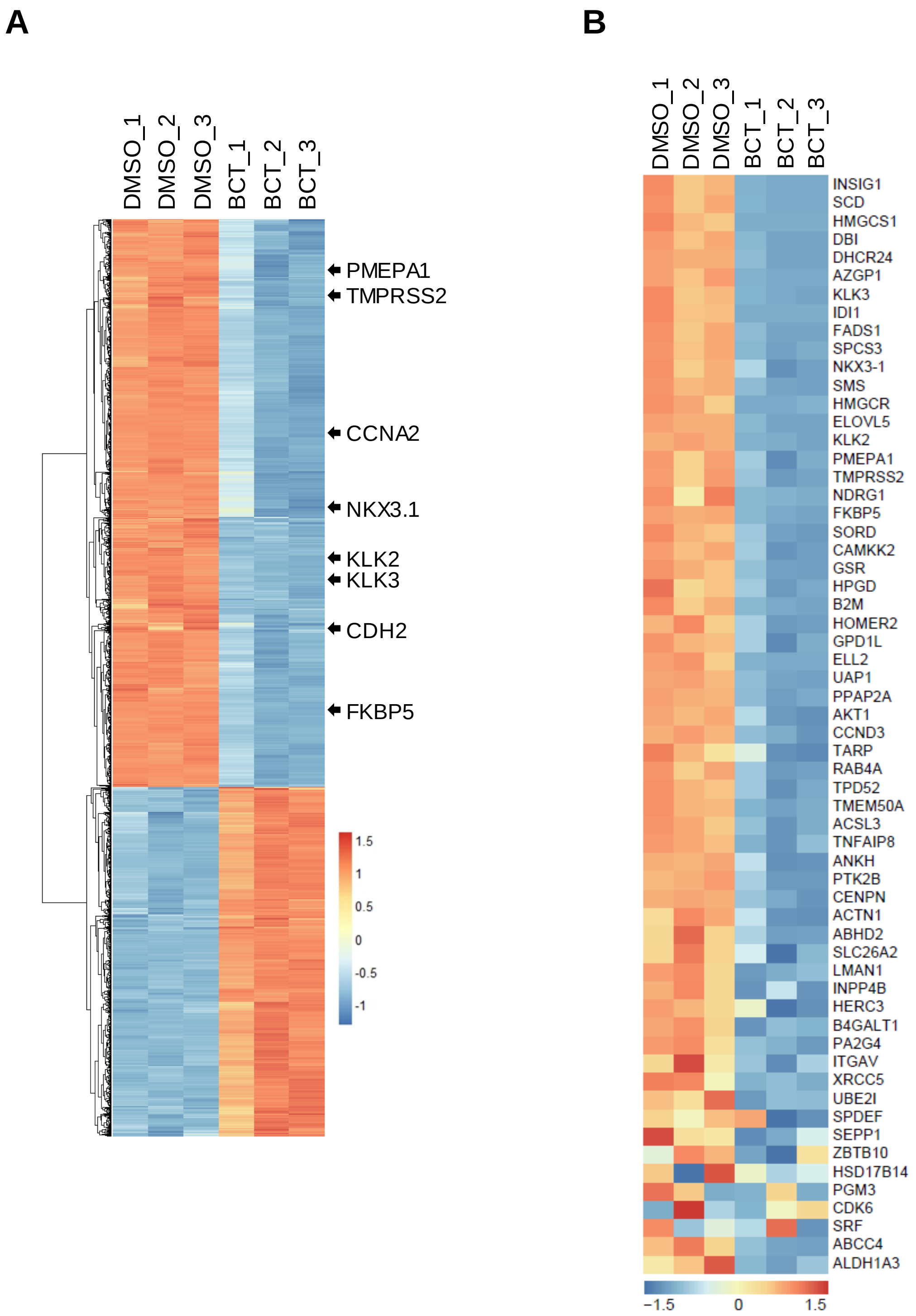
A
DMSO_1
DMSO_2
DMSO_3
BCT_1
BCT_2
BCT_3
PMEPA1
TMPRSS2
CCNA2
NKX3.1
KLK2
KLK3
CDH2
FKBP5
1.5
1
0.5
0
-0.5
-1
B
DMSO_1
DMSO_2
DMSO_3
BCT_1
BCT_2
BCT_3
INSIG1
SCD
HMGCS1
DBI
DHCR24
AZGP1
KLK3
IDI1
FADS1
SPCS3
NKX3-1
SMS
HMGCR
ELOVL5
KLK2
PMEPA1
TMPRSS2
NDRG1
FKBP5
SORD
CAMKK2
GSR
HPGD
B2M
HOMER2
GPD1L
ELL2
UAP1
PPAP2A
AKT1
CCND3
TARP
RAB4A
TPD52
TMEM50A
ACSL3
TNFAIP8
ANKH
PTK2B
CENPN
ACTN1
ABHD2
SLC26A2
LMAN1
INPP4B
HERC3
B4GALT1
PA2G4
ITGAV
XRCC5
UBE2I
SPDEF
SEPP1
ZBTB10
HSD17B14
PGM3
CDK6
SRF
ABCC4
ALDH1A3
−1.5
0
1.5

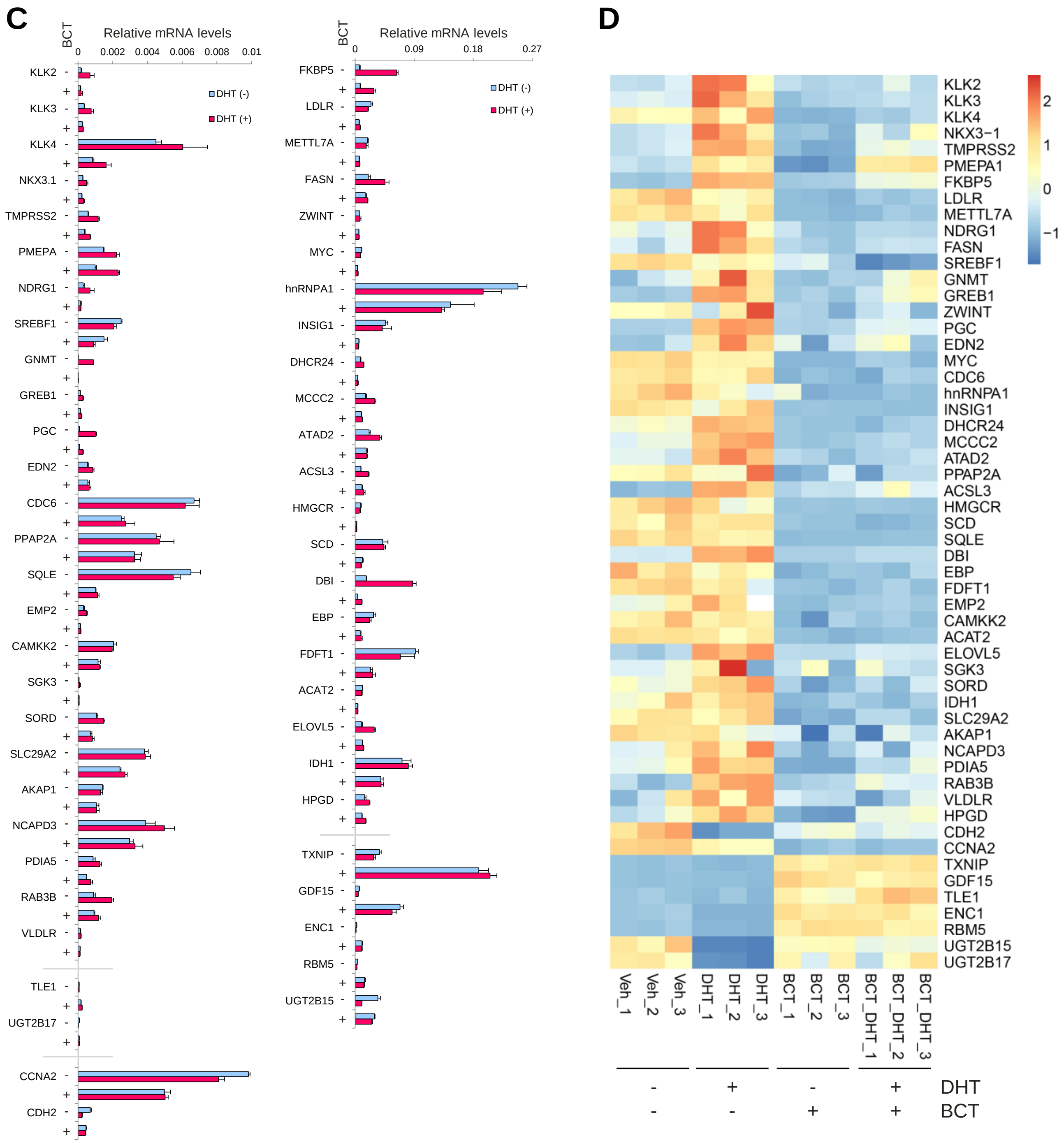

Figure S3. BCT-regulated genes in 22RV1 cells. (A) Hierarchical clustering of the differentially expressed genes between vehicle (DMSO) and 10 nM BCT treatment in 22RV1 cells with fold change (FC) > 1.8 and FDR < 0.05. (B) Heat map of mRNA levels of BCT-repressed androgen target genes listed in the Hallmark_androgen response gene set. (C,D) Validation of BCT-regulated AR-FL target genes by qRT-PCR in 22RV1 cells cultured in hormone-depleted medium in the absence or presence of 10 nM DHT. qRT-PCR data are presented as means ± s.d. (n=3) (C), and the visualized heatmap was generated using R package based on the qRT-PCR data (D).

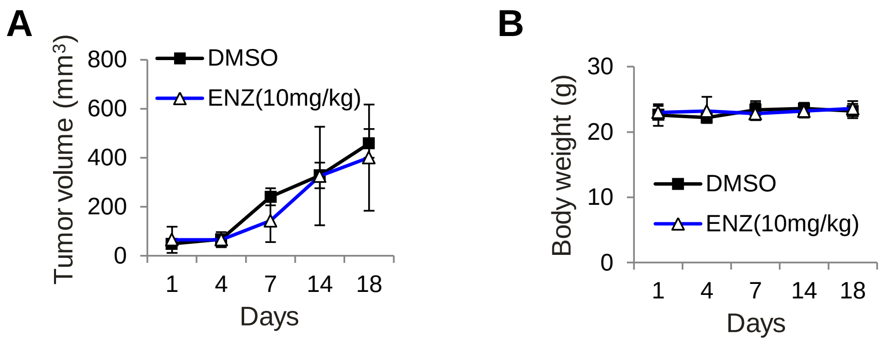

Figure S4. 22RV1 xenograft tumors are resistant to ENZ. 22RV1 cells were injected into the right flank of BALB/c nude mice. Mice bearing 22RV1 xenograft tumor were treated i.p. with DMSO or ENZ (10 mg/kg) everyday for 18 days (n= 5). Tumor growth curves are shown (A), and mouse body weight was measured (B).

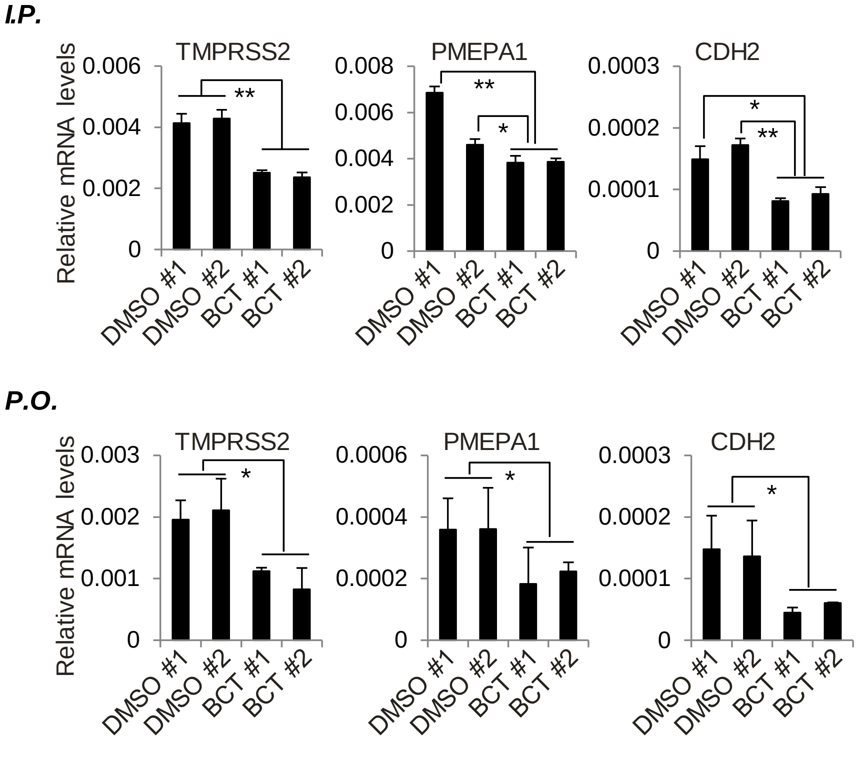

Figure S5. BCT inhibits in vivo expression of AR-FL/AR-V7 target genes. qRT-PCR analysis of TMPRSS2, PMEPA1, and CDH2 expression in 22RV1 xenograft tumors treated with DMSO or BCT (2 mg/kg). Data are means ± s.d. (n = 3). $^{**}p < 0.01$, $^{*}p < 0.05$.

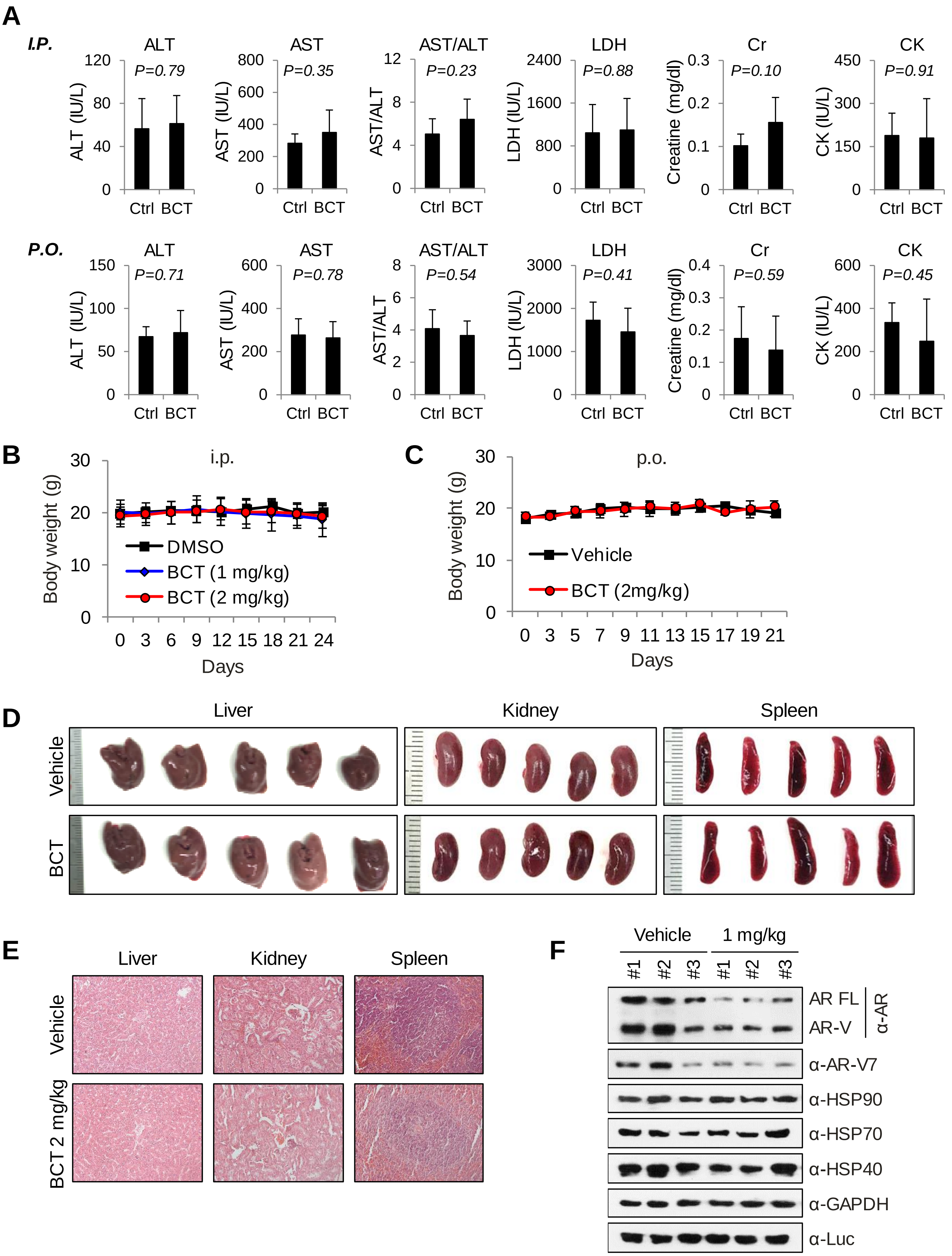
A
I.P.
ALT
P=0.79
ALT (IU/L)
120
80
40
0
Ctrl BCT
AST
P=0.35
AST (IU/L)
800
600
400
200
0
Ctrl BCT
AST/ALT
P=0.23
AST/ALT
12
8
4
0
Ctrl BCT
LDH
P=0.88
LDH (IU/L)
2400
1600
800
0
Ctrl BCT
Cr
P=0.10
Creatine (mg/dl)
0.3
0.2
0.1
0
Ctrl BCT
CK
P=0.91
CK (IU/L)
450
300
150
0
Ctrl BCT
P.O.
ALT
P=0.71
ALT (IU/L)
150
100
50
0
Ctrl BCT
AST
P=0.78
AST (IU/L)
600
400
200
0
Ctrl BCT
AST/ALT
P=0.54
AST/ALT
8
6
4
2
0
Ctrl BCT
LDH
P=0.41
LDH (IU/L)
3000
2000
1000
0
Ctrl BCT
Cr
P=0.59
Creatine (mg/dl)
0.4
0.3
0.2
0.1
0
Ctrl BCT
CK
P=0.45
CK (IU/L)
600
400
200
0
Ctrl BCT
B
i.p.
Body weight (g)
30
20
10
0
DMSO
BCT (1 mg/kg)
BCT (2 mg/kg)
0 3 6 9 12 15 18 21 24
Days
C
p.o.
Body weight (g)
30
20
10
0
Vehicle
BCT (2mg/kg)
0 3 5 7 9 11 13 15 17 19 21
Days
D
Liver
Kidney
Spleen
Vehicle
BCT
E
Liver
Kidney
Spleen
Vehicle
BCT 2 mg/kg
F
Vehicle
1 mg/kg
#1 #2 #3 #1 #2 #3
AR FL
AR-V
α-AR
α-AR-V7
α-HSP90
α-HSP70
α-HSP40
α-GAPDH
α-Luc

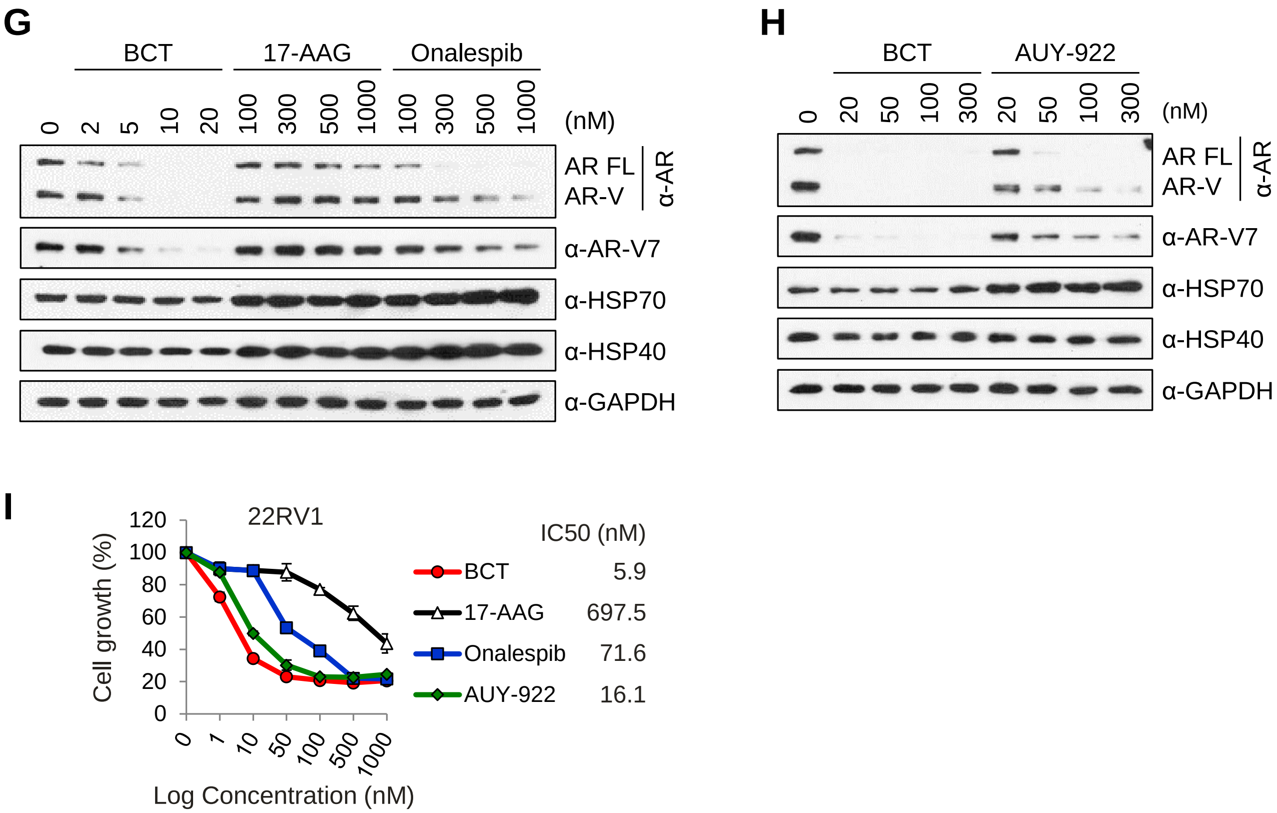

Figure S6. BCT does not have apparent in vivo toxicity but downregulates protein levels of AR-FL and AR-V7. (A) No significant differences were observed in the levels of plasma enzymes including alanine transaminase (ALT), aspartate transaminase (AST), lactate dehydrogenase (LDH), creatinine (CR), and creatine phosphokinase (CPK) between the control and BCT treatment groups. (B, C) BCT does not affect the body weight of mice. Mice bearing 22RV1 xenograft tumor were treated i.p. (B) or p.o. (C) with BCT, and mouse body weight was measured every 3 days (i.p.) or every the other day (p.o.). (D, E) Mice livers, kidneys, and spleens from the same experiment as Fig. 3E were anatomically (D) and histologically (E, H&E staining) evaluated. (F) Immunoblot analyses of AR-FL, AR-V7, and HSPs in 22RV1 xenograft tumors treated i.p. with DMSO or BCT. (G, H) Effect of BCT and first- (17-AAG) and second-generation (onalespib and AUY-922) HSP90 inhibitors on the expression of HSP70 and HSP40 and stability of AR-FL/AR-V7 in 22RV1 cells. The protein levels of AR-FL, AR-V7, HSP70, HSP40, and GAPDH were determined by immunoblot analysis. BCT decreased protein levels of AR-FL and AR-V7 more effectively than 17-AAG, onalespib, and AUY-922, without affecting the expression of inducible HSPs, HSP70 and HSP40. The ranges of concentrations of HSP90 inhibitors tested: 2~20 nM or 20~300 nM for BCT; 100~1000 nM for 17-AAG and onalespib; 20~300 nM for AUY-922 (luminespib). (I) 22RV1 cells were treated with different concentrations of indicated compounds. Cell proliferation was detected by MTT assays, and IC50 values were determined. Data are means ± s.d. (n=6).

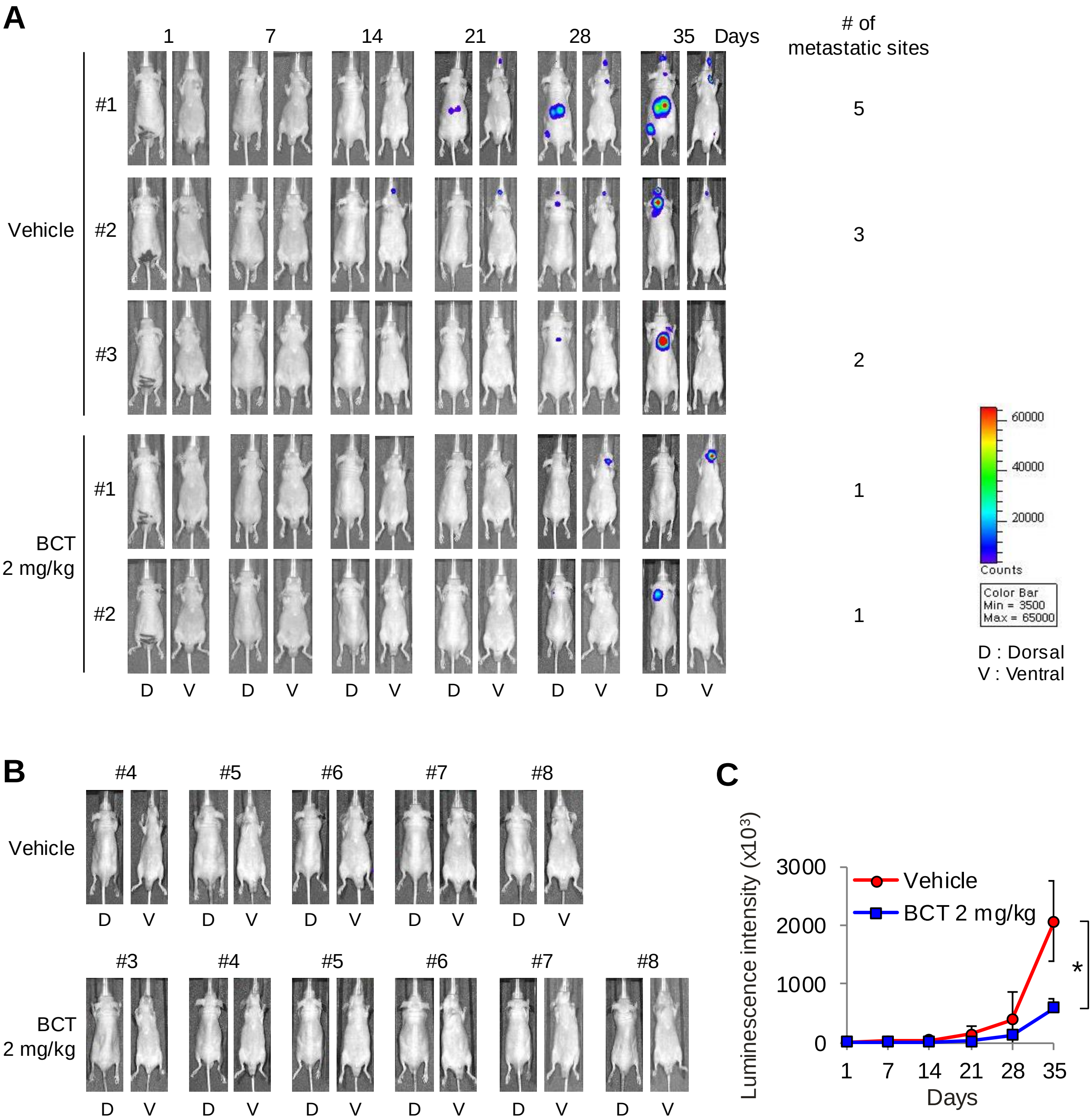

Figure S7. BCT inhibits the growth and metastasis of CRPC cells in vivo. 22RV1-LUC-T cells were intracardially injected in male nude mice, and the mice were treated with vehicle or BCT (2 mg/kg) by oral gavage every other day for 7 weeks (n= 8). Bioluminescence images were taken from both the dorsal and ventral sides of the mice every week. (A) Bioluminescence images of mice bearing metastatic tumors and numbers of metastatic sites. (B) Bioluminescence images of non-metastatic mice at day 35. (C) Normalized bioluminescence of metastatic tumors of the control and BCT groups. BCT treatment induced 72% reduction in bioluminescence signals of metastatic tumors at the end of treatment. *$p < 0.05$.

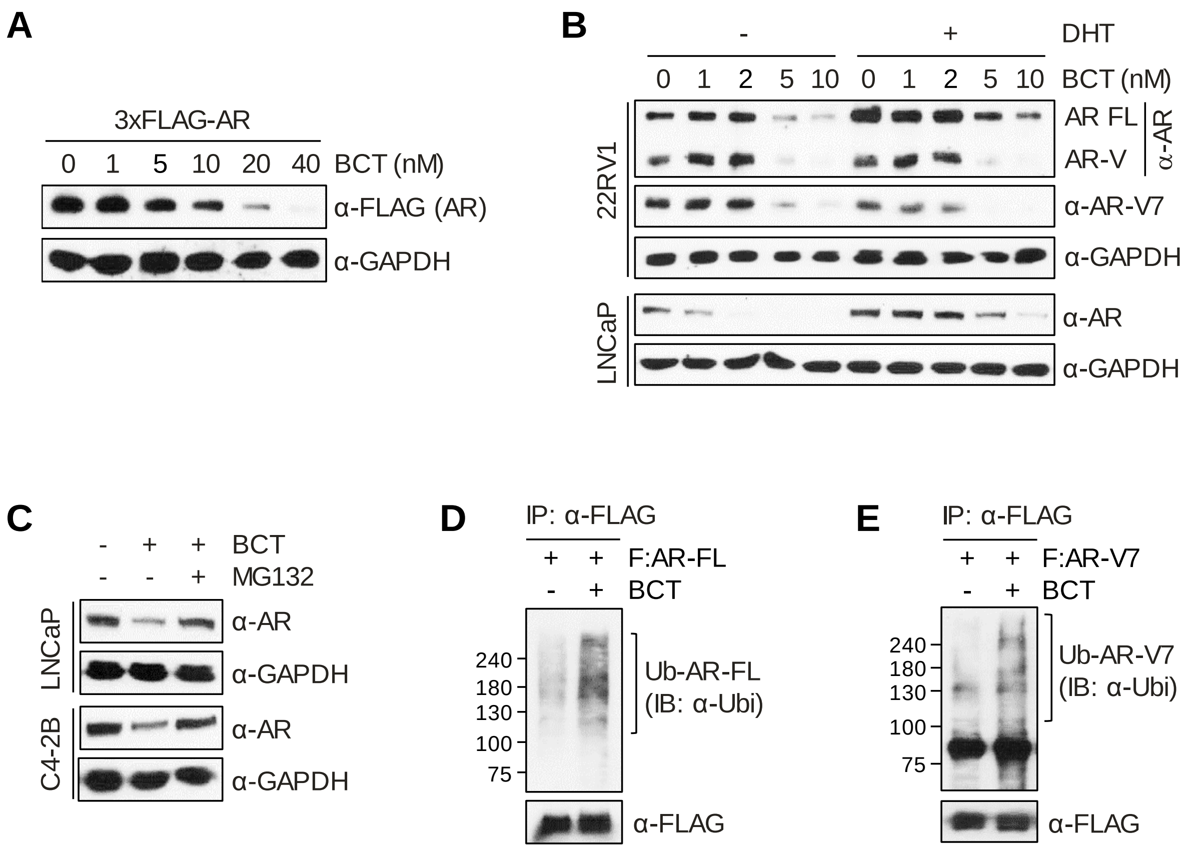

Figure S8. BCT negatively regulates protein stability of AR-FL and AR-V7. (A) 293T cells transfected with 3xFLAG-AR-FL expression vector were treated with indicated concentrations of BCT for 24 hr, and cell lysates were analyzed by immunoblot with indicated antibodies. (B) 22RV1 and LNCaP cells were treated with 20nM DHT and indicated concentrations of BCT as indicated for 24 hr. Cell lysates were analyzed by immunoblot with indicated antibodies. (C) LNCaP and C4-2B cells were treated with 10nM BCT and 10 μM MG132 as indicated for 24 hr, and cell lysates were analyzed by immunoblot with indicated antibodies. (D, E) 22RV1 cells transfected with 3xFLAG-AR-FL (D) or 3xFLAG-AR-V7 (E) expression vector were treated with 0.1 nM BCT and 10 μM MG132 as indicated for 8 hr. Cell lysates were immunoprecipitated with anti-FLAG M2 agarose and immunoblotted with indicated antibodies.

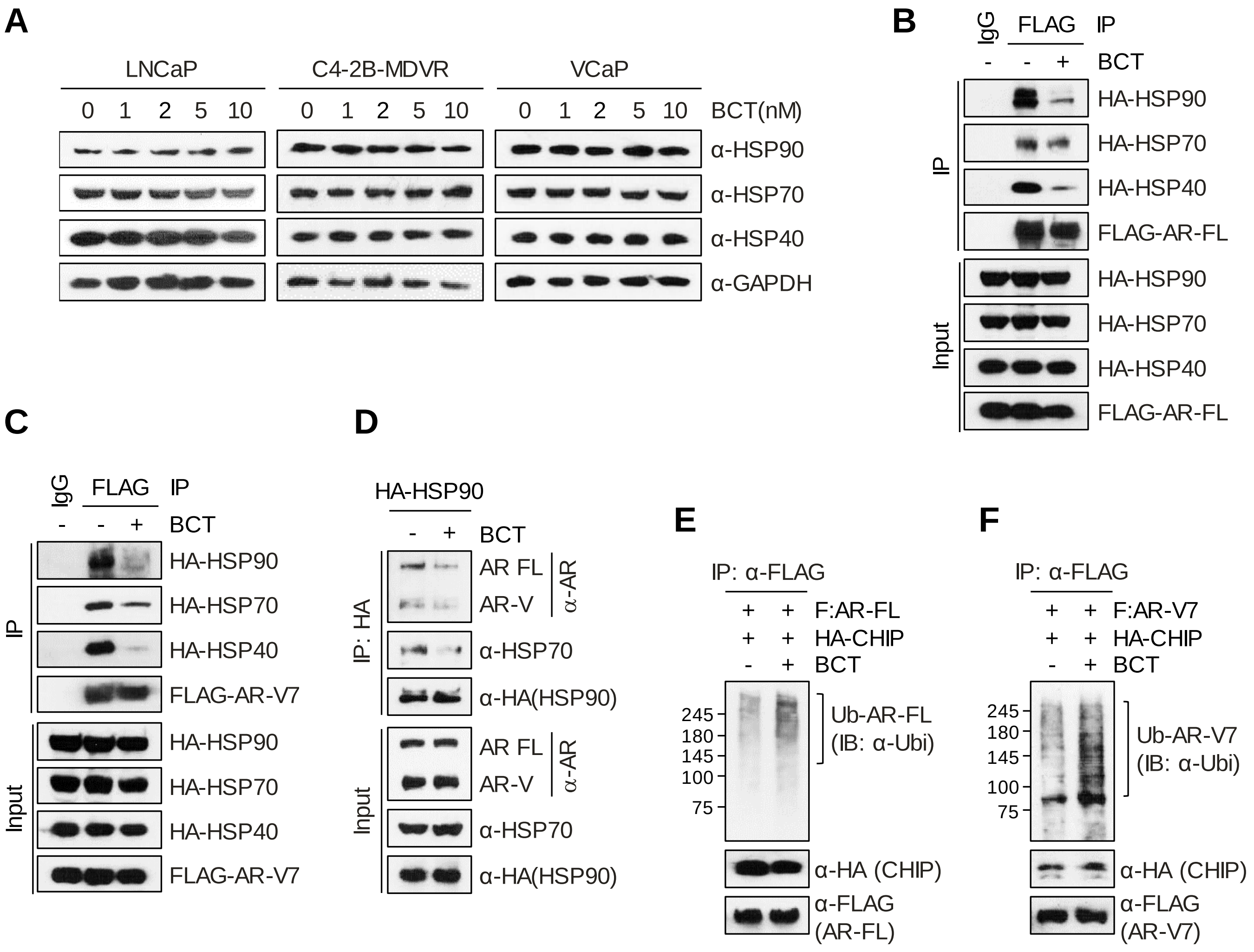

Figure S9. BCT inhibits interactions between AR-FL/AR-V7 and HSPs and increases CHIP-mediated ubiquitination of AR-FL and AR-V7. (A) LNCaP, C4-2B-MDVR, and VCaP cells were treated with indicated concentrations of BCT for 24 hr, and cell lysates were analyzed by immunoblot using the indicated antibodies. (B, C) 293T cells transfected with HA-tagged HSP expression vectors and 3xFLAG-AR-FL (B) or 3xFLAG-AR-V7 (C) expression vector were treated with 10 μM MG132 and DMSO or 10 nM BCT for 12 hr. Cell lysates were immunoprecipitated with anti-FLAG M2 agarose and immunoblotted with indicated antibodies. (D) 22RV1 cells transfected with HA-tagged HSP90 expression vector were treated with 10 μM MG132 and DMSO or BCT for 4 hr, and cell lysates were immunoprecipitated with anti-HA agarose and immunoblotted with indicated antibodies. (E, F) 293T cells transfected with HA-CHIP and 3xFLAG-AR-FL (E) or 3xFLAG-AR-V7 (F) expression vectors were treated with 10 μM MG132 and DMSO or BCT for 8 hr. Cell lysates were immunoprecipitated with anti-FLAG M2 agarose and immunoblotted with indicated antibodies.

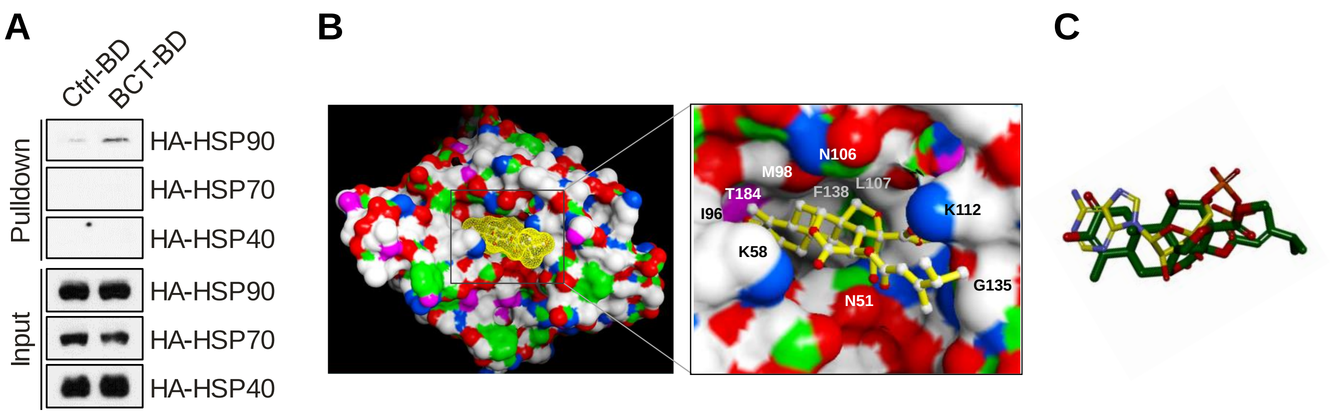

Figure S10. BCT binds to HSP90. (A) 293T cell extracts expressing HA-HSP90, HA-HSP70, and HA-HSP40 were incubated with BCT-immobilized Unosphere beads or control Unosphere beads, and bound proteins were analyzed by immunoblot with anti-HA antibody. (B) Molecular docking model of BCT bound to HSP90. BCT (shown in ball-and-stick representation) fits into the ATP-binding pocket of HSP90. Amino acid residues involved in BCT binding in HSP90 are labelled. (C) Docked conformation of BCT (green carbon) in comparison with that of ADP (yellow carbon).

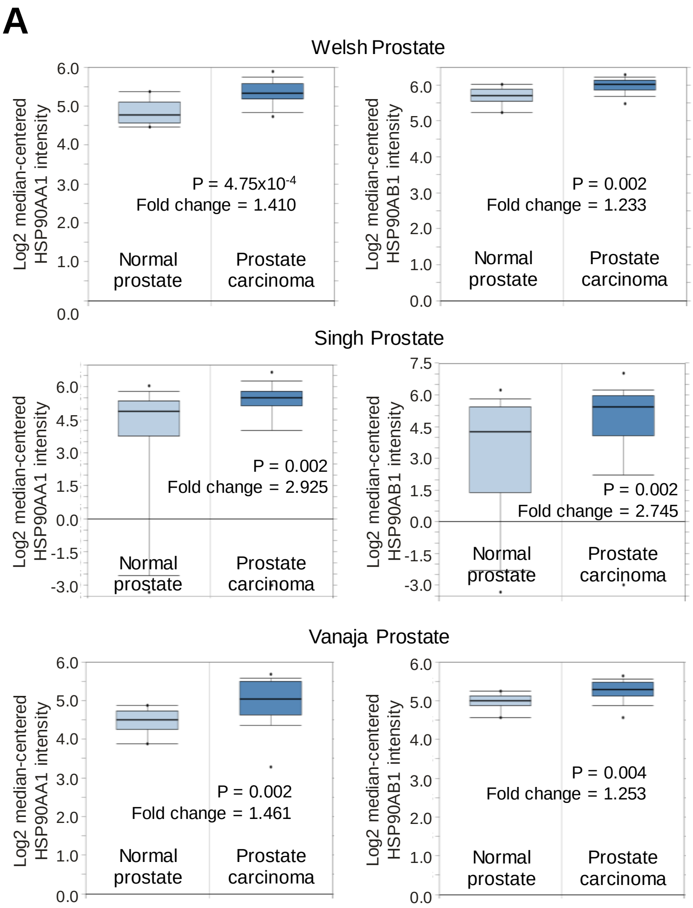

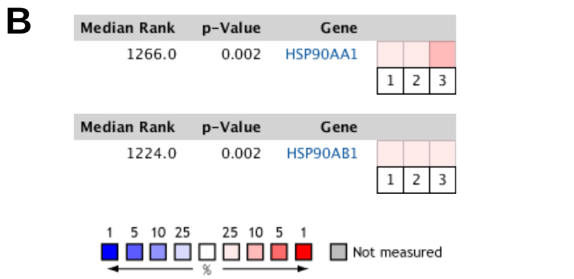

Figure S11. HSP90 expression is upregulated in PCa tumors. (A) Analysis of HSP90AA1 and HSP90AB1 mRNA expression in human PCa using databases at Oncomine (http://www.oncomine.org). P-value was determined by Student's t-test. (B) Meta-analyses of HSP90AA1 and HSP90AB1 gene expression from three Oncomine databasets where colored squares indicate the median rank for HSP90AA1 and HSP90AB1 (vs. Normal tissue) across 3 analyses. Oncomine datasets: 1. Singh Prostate; 2. Vanaja Prostae; 3. Welsh Prostate.

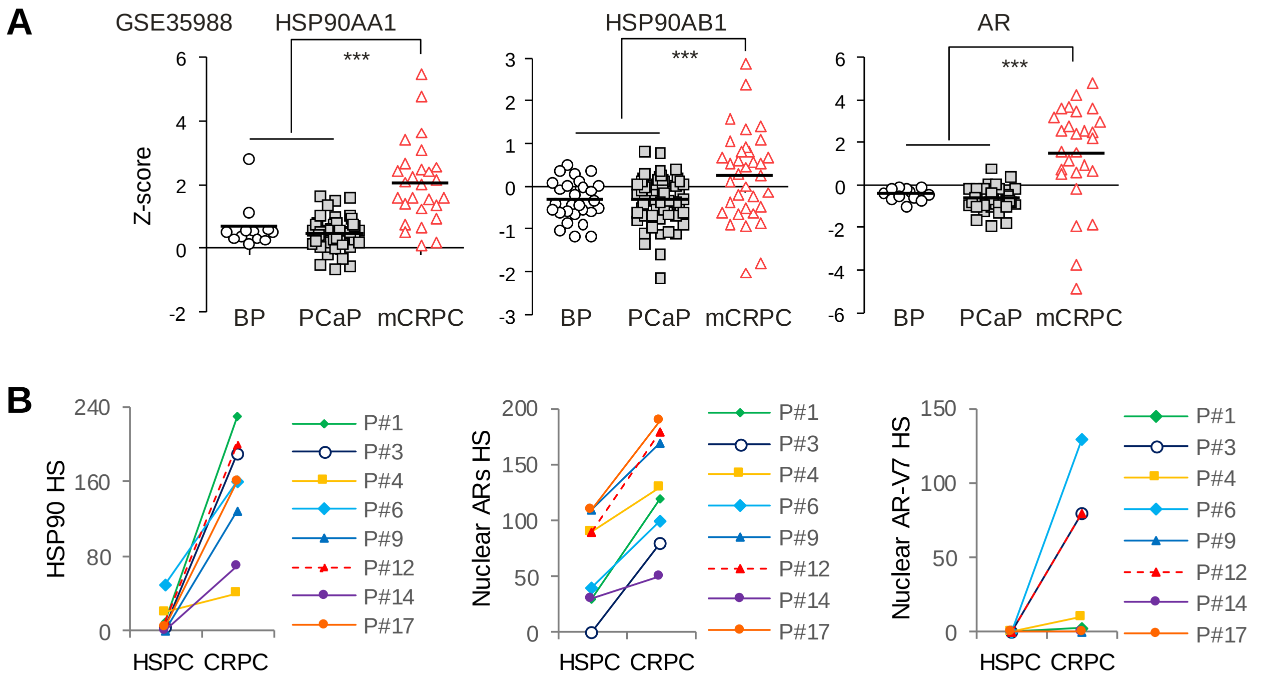

Figure S12. HSP90 expression is correlated with PCa progression and with levels of AR/AR-V7. (A) HSP90 level is correlated with PCa progression. HSP90 gene (HSP90AA1 and HSP90AB1) expression levels were determined in benign prostate (BP), primary PCa (PCaP), and metastatic CRPC (mCRPC). Statistical analysis was performed using one-way ANOVA. ***p < 0.001. (B) Protein expression (H-score, HS) of HSP90 and nuclear ARs and AR-V7 in matched HSPC and CRPC tissues.

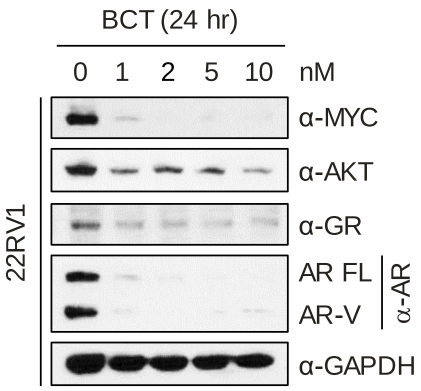

Figure S13. BCT downregulates protein levels of MYC, AKT, and GR in CRPC cells. 22RV1 cells were treated with indicated concentrations of BCT for 24h, and cell lysates were analyzed by immunoblot with indicated antibodies.

Table S1. List of compounds screened in this study.

No	Compounds	Manufacturer	Cat.
1	Ailanthone	TOCRIS	6161
2	Enzalutamide	Biovision	9479-10
3	Artesunate	abcam	ab142829
4	Artemisinin	abcam	ab141308
5	Dihydroartemisinin	abcam	ab142690
6	Artemether	abcam	ab142689
7	Chloroquine diphosphate	Santa cruz	sc-205629
8	Niclosamide	abcam	ab120868
9	Primaquine diphosphate	abcam	ab142355
10	Plumbagin	abcam	ab145184
11	Pyrimethamine	abcam	ab143106
12	Sulfadoxine	abcam	ab143199
13	Hydroxychloroquine sulfate	Santa cruz	sc-215157
14	Amodiaquine dihydrochloride dihydrate	Selleckchem	S4589
15	Lumefantrine	Santa cruz	sc-207836
16	Mefloquine Hydrochloride	Santa cruz	sc-211784
17	10,11-Dehydrocurvularin	abcam	ab144855
18	Astemizole	Santa cruz	sc-201088
19	Licochalcone A	Selleckchem	S7828
20	Quinine hydrochloride	TOCRIS	4114
21	Magnolol	Selleckchem	S2321
22	Andrographolide	Santa cruz	sc-205594
23	Phlorizin	TOCRIS	4627
24	Ganoderic acid A	Selleckchem	S4753
25	Bruceine A	ChemNorm	TRY0101
26	1,8-Dihydroxyanthraquinone	ChemNorm	TPC0344
27	1,8-Diacetoxy-3-carboxyanthraquinone	ChemNorm	TPC0250
28	Eurycomanone	ChemNorm	TBZ1340
29	Aurantio-obtusin	ChemNorm	TB0101-0100
30	Mollugin	ChemNorm	TB0623-0500
31	β，β-Dimethylacrylalkannin	ChemNorm	TB0635-0100
32	Acetylshikonin	ChemNorm	TB0586-0100
33	Aloin A	ChemNorm	TB0786-0025
34	Sennoside A	ChemNorm	TB0027-1000
35	Cryptotanshinone	ChemNorm	TB0061
36	Rhein	ChemNorm	TB0043
37	Brusatol	SIGMA	SML1868
38	Quassin	ChemFacs	CFN97246-1MG
39	Curvularin	ENZO	ALX-380-305-M001
		TRC	B6893109
40	Bruceantin	ChemNorm	TRY0213
		JH Chem	JH41451756-100MG
41	Yadanzioside I	ChemFacs	CFN96421-1MG
42	Yadanzioside F	ChemFacs	CFN96423-1MG
43	Yadanzioside C	ChemFacs	CFN96424-1MG
44	Bruceantinol	ChemFaces	CFN89336-1MG
45	Bruceanic acid C	ChemFaces	CFN91975-1MG
46	Bruceine D	ChemFaces	CFN90771-10-MG
47	Dehydrobruceine A	ChemFaces	CFN89341- 1MG
48	Bruceantinoside A	ChemFaces	CFN-89343-1MG
49	(+)-Glaucarubinone	ChemFaces	CFN92979-1MG
50	Yadanziolide A	ChemFaces	CFN96428-1MG
51	Yadanziolide B	ChemFaces	CFN96427-MG
52	Yadanziolide C	ChemFaces	CFN96426-1MG

Table S2. Screening of compounds for inhibitory activity against AR-V7 (DHT-) and AR-FL (DHT+) transcriptional activities in 22RV1 cells.

Compound screening at 10 μM

		MMTV-LUC acvitivity (%)	
Conc.	Compound	DHT-	DHT+
	-	22.3±5.2	100.0±8.7
10 μM	Enzalutamide	17.0±7.4	51.8±0.1
	Ailanthone	1.9±0.5	2.2±0.4
	Artesunate	23.1±0.2	117.5±9.3
	Artemisinin	22.9±0.9	156.1±17.6
	Dihydroartemisinin	24.7±2.2	137.5±7.0
	Artemether	20.9±4.2	138.2±8.8
	Chloroquine diphosphate	18.5±0.8	106.9±1.0
	Niclosamide	2.0±0.1	2.3±0.5
	Primaquine diphosphate	24.4±3.8	115.6±7.0
	Plumbagin	5.6±1.6	7.1±0.1
	Pyrimethamine	28.0±0.0	149.2±2.4
	Sulfadoxine	24.9±0.9	170.3±11.2
	Hydroxychloroquine sulfate	19.8±0.4	122.1±0.7
	Amodiaquine dihydrochloride dihydrate	19.6±0.6	102.2±4.3
	Lumefantrine	29.5±2.0	160.7±18.2
	Mefloquine Hydrochloride	24.5±4.3	109.0±6.3
	10,11-Dehydrocurvularin	3.5±0.5	5.5±0.6
	Astemizole	13.1±1.1	59.5±15.5
	Licochalcone A	43.5±1.5	235.6±0.2
	Quinine hydrochloride	31.1±1.4	156.9±2.3
	magnolol	27.8±2.6	151.9±8.6
	andrographolide	33.7±0.9	196.2±5.0
	Phlorizin	33.1±1.4	184.4±12.6
	Ganoderic acid A	30.0±0.8	148.9±2.8
	Bruceine A	1.0±0.1	1.0±0.1
	1,8-Dihydroxyanthraquinone	21.9±2.6	168.1±32.4
	1,8-Diacetoxy-3-carboxyanthraquinone	27.2±1.2	114.5±13.7
	Eurycomanone	3.2±0.3	4.2±0.7
	Aurantio-obtusin	44.6±7.1	128.6±13.6
	Mollugin	22.0±2.1	146.2±19.7
	β,β-Dimethylacrylalkannin	3.0±0.6	8.1±1.0
	Acetylshikonin	2.1±0.1	3.5±0.1
	Aloin A	26.5±5.3	123.0±12.7
	Sennoside A	30.8±5.7	136.5±8.8
	Cryptotanshinone	13.1±4.1	67.2±11.9
	Rhein	25.6±1.5	122.7±5.7
	Brusatol	1.0±0.2	1.2±0.2
	Quassin	31.0±4.9	102.7±13.4
	Curvularin	18.0±0.7	91.6±9.4
	Bruceantin	4.9±0.7	3.9±0.4
	Yadanzioside C	32.7±3.4	5.8±0.7
	Yadanzioside F	39.6±6.4	139.5±11.0
	Yadanzioside I	36.5±3.1	136.0±7.1
	Bruceantinol	13.0±10.0	44.5±2.1
	Bruceanic acid C	11.0±1.0	85.8±14.2
	Bruceine D	1.2±0.2	1.1±0.1
	Bruceantinoside A	1.0±0.1	3.8±1.1
	Dehydrobruceine A	20.1±2.2	164.2±16.0
	(+)-Glaucarubinone	18.3±4.5	117.7±3.5
	Yadanziolide A	1.4±0.1	4.1±0.2
	Yadanziolide B	1.4±0.2	4.0±0.5
	Yadanziolide C	0.9±0.1	1.1±0.1

Compound screening at 1 μM

		MMTV-LUC acvitivity (%)	
Conc.	Compound	DHT-	DHT+
	-	25.0±5.0	100.0±11.4
1 μM	Enzalutamide	20.5±2.7	96.7±1.7
	Ailnahtone	4.2±0.6	4.5±0.9
	Niclosamide	27.9±2.5	56.7±5.3
	Plumbagin	59.9±4.5	142.9±27.5
	10,11-Dehydrocurvularin	106.8±6.9	206.9±15.0
	Astemizole	32.0±1.5	126.4±3.6
	Bruceine A	3.7±0.5	3.6±0.2
	Eurycomanone	16.0±2.1	51.3±4.6
	β,β-Dimethylacrylalkannin	27.1±3.1	107.9±7.0
	Acetylshikonin	27.8±2.1	113.0±16.6
	Brusatol	3.8±0.4	4.0±0.5
	Bruceantin	4.5±0.5	4.4±0.3
	Yadanzioside C	35.3±5.4	55.6±0.7
	Bruceantinol	11.8±1.9	118.9±21.8
	Bruceine D	1.0±0.2	2.0±0.2
	Bruceantinoside A	1.7±0.8	8.0±0.2
	Yadanziolide A	9.2±2.2	58.3±10.9
	Yadanziolide B	1.2±0.1	4.2±0.4
	Yadanziolide C	2.6±0.5	12.1±2.0

Compound screening at 0.1 μM

		MMTV-LUC acvitivity (%)	
Conc.	Compound	DHT-	DHT+
	-	25.7±3.1	100.0±3.0
0.1 μM	Ailanthone	7.3±0.5	9.8±0.4
	Bruceine A	5.6±0.5	5.7±1.0
	Eurycomanone	34.6±3.2	107.7±4.7
	Brusatol	5.5±0.9	5.6±0.6
	Bruceantin	4.0±0.7	4.0±0.5
	Bruceine D	6.9±1.0	32.6±6.2
	Bruceantinoside A	6.7±0.8	61.0±7.3
	Yadanziolide B	3.9±0.9	32.8±9.0
	Yadanziolide C	12.1±0.4	92.5±0.9

22RV1 cells transfected with MMTV-LUC were treated with indicated compounds at indicated concentrations with or without 20 nM DHT for 24 hr. Luciferase activities were measured, and results were expressed as the ratio of luciferase activity. Data are means±s.d. (n=3).

Table S3. qRT-PCR primer sets used in this study.

β-actin	Forward: CCA CAC TGT GCC CAT CTA CG<br>Reverse: AGG ATC TTC ATG AGG TAG TCA GTC AG
GAPDH	Forward: TCT GGT AAA GTG GAT ATT GTT GCC<br>Reverse: GAA GAT GGT GAT GGG ATT TCC
KLK2	Forward: GCT GCC CAT TGC CTA AAG AAG<br>Reverse: TGG GAA GCT GTG GCT GAC A
KLK3 (PSA)	Forward: TGT GTG CTG GAC GCT GGA<br>Reverse: CAC TGC CCC ATG ACG TGA T
TMPRSS2	Forward: CCT GCA GGG ACA TGG GCT ATA<br>Reverse: CCG GCA CTT GTG TTC AGT TTC
FKBP5	Forward: AGG CTG CAA GAC TGC AGA TC<br>Reverse: CTT GCC CAT TGC TTT ATT GG
NKX3.1	Forward: CCG AGC CAG AAA GGC ACT TGG G<br>Reverse: AGC GCT TCT GCG GCT GCT TA
CDH2	Forward: GGA GAT CCT ACT GGA CGG TTC G<br>Reverse: CCC TTG GCT AAT GGC ACT TGA
CCNA2	Forward: CGC TGG CGG TAC TGA AGT C<br>Reverse: GAG GAA CGG TGA CAT GCT CAT
AR-FL	Forward: ATC TGT GGA GAT GAA GCT TCT<br>Reverse: GGG CTG ACA TTC ATA GCC TTC AAT GTG TGA C
AR-V7	Forward: ATC TGT GGA GAT GAA GCT TCT<br>Reverse: TGC CAA CCC GGA ATT TTT CTC CC
PMEPA1	Forward: TGC AAC TGC AAA CGC TCT TT<br>Reverse: CCA CCA CCA TCA CCA TCA TC
KLK4	Forward: GGC ACT GGT CAT GGA AAA CGA<br>Reverse: TCA AGA CTG TGC AGG CCC AGC C
LDLR	Forward: CAA TGT CTC ACC AAG CTC TGG<br>Reverse: CAT CTG TCT CGA GGG GTA GCT
METTL7A	Forward: CAG AGT GCT GAG ACC GGG A<br>Reverse: CTG GTC AGG TTG CAC CCA TC
NDRG1	Forward: ACA ACC CTG AGA TGG TGG AG<br>Reverse: TGT GGA CCA CTT CCA CGT TA
FASN	Forward: CTG GCT CAG CAC CTC TAT CCC<br>Reverse: CAG GTT GTC CCT GTG ATC
SREBF1	Forward: GCT TCT CTA CAG GAA GCC CTC<br>Reverse: TCT GCC TTG ATG AAG TGG GGC
GNMT	Forward: AGT ACA AGG CAT GGC TGC TT<br>Reverse: CTT TGC AGT CTG GCA AGT GA

Table S3. qRT-PCR primer sets used in this study (*continued*).

GREB1	Forward: CAA AGA ATA ACC TGT TGG CCC TGC<br>Reverse: GAC ATG CCT CCG CTC TCA TAC TTA
ZWINT	Forward: CCC TGA CTC AGA TGG AGG AA<br>Reverse: AAA CCT CCG CCA GAT GCT
PGC	Forward: ACA GGC ACC TCT CTG CTC ACT<br>Reverse: AGT AGC CGT TGT TAC TGA GGA T
EDN2	Forward: TCT GGG TGA ACA CTC CTG AAC A<br>Reverse: GAA GGC AGA AGG TGG CAC AG
MYC	Forward: CCT CAA CGT TAG CTT CAC CAA<br>Reverse: CTC CTC GTC GCA GTA GAA ATA
CDC6	Forward: AAA GAG AAT GGT CCC CCT CAC TC<br>Reverse: AGT TTT TCC AGT TCC AGG AGC AC
hnRNPA1	Forward: TTG GAG GTG GTG GAA GCT ACA<br>Reverse: ATA GCT ACT GCT GCT GCT GGA
INSIG1	Forward: ATT TTT CTC AGG AGG CGT CA<br>Reverse: CTG CGG GTT GGT AAT TGA GT
DHCR24	Forward: GCC GCT CTC GCT TAT CTT CG<br>Reverse: GTC TTG CTA CCC TGC TCC TT
MCCC2	Forward: TTT GTC CAG TTA TGC TGC CAA AGA<br>Reverse: GGC ACC ATC CTT GGC AAT TC
ATAD2	Forward: CAC CGA GTA CTC CTG TGG CTT<br>Reverse: TCT AGC TCG AGT CAT TCG CAG
PPAP2A	Forward: CCA CAC TGC AAT TTG GTC TTG<br>Reverse: CCC TGA ATG AGT CCA GTC AAC
ACSL3	Forward: GGG CCA AAG TGT GAC AAT GG<br>Reverse: CGG GTT CAA ACT CTC CAA TAT CC
HMGCR	Forward: GCC TGA CAC AGG AAC CTG AA<br>Reverse: AGT GCT GTC AAA TGC CTC CTZ
SCD	Forward: CGA CGT GGC TTT TTC TTC TC<br>Reverse: CCT TCT CTT TGA CAG CTG GG
SQLE	Forward: CAG CAA GCT TCC TTC CTC CTT<br>Reverse: CTC CAC CAG TAA GTG GAT GCC
DBI	Forward: AAG CAA CTG TGG GCG ACA TAA<br>Reverse: CTC ATT CCA GGC ATC CCA CTT
EBP	Forward: GGT TGT CCC ATT GGG GAC TTG<br>Reverse: GGC AAG CTG TGA TGG TTT CCA
FDFT1	Forward: GCA ACG CAG TGT GCA TAT TTT<br>Reverse: CGC CAG TCT GGT TGG TAA AGG

Table S3. qRT-PCR primer sets used in this study (*continued*).

EMP2	Forward: TCT TCG TGC TCC AGC TCT TCC<br>Reverse: GAG TAG CCG TAG CTG CCT TCT
CAMKK2	Forward: TCC AGA CCA GCC CCG ACA TAG<br>Reverse: CAG GGG TGC AGC TTG ATT TC
ACAT2	Forward: TGG GCA GAA TCC TGT TAG ACA<br>Reverse: TGC AAG GCA CAC AGC TTT TAG
ELOVL5	Forward: ATT GCT AGG CCC TCG AGA TAC<br>Reverse: AAA TCC CCC GGC AAG AGA ATG
SGK3	Forward: CCA GGA GTG AGT CTT ACA G<br>Reverse: CCA GCC ACA TTA GGA TTA
SORD	Forward: TGA GAT GAC CAC CGT ACC CC<br>Reverse: GAA ACA CGC CCT TGA TAT CCA
IDH1	Forward: TGC AAA AAT ATC CCC CGG CTT<br>Reverse: TTT GGG TTC CGT CAC TTG GTG
SLC29A2	Forward: CAA CAT CAT GGA CTG GCT GGG<br>Reverse: CAT GAA GAG GGG CAC GAA CAG
AKAP1	Forward: GGA GTT GTC AGA CTT GGG GGC<br>Reverse: CTG CCT GGG CAT TTT GGA AGC
NCAPD3	Forward: GCA TCT GCA GAG ACA CCA GCA<br>Reverse: GAG AAC GAT GTT GGA GAG GCG
PDIA5	Forward: TGG AGT GGC TGA AGA ATC CGC<br>Reverse: TCA GGT GAT AAA CGG AGC CGC
RAB3B	Forward: TAC CGG ACC ATC ACA ACA GCC<br>Reverse: GTC ACA CTT GTT CCC CAC CAG
VLDLR	Forward: TCA GTG TAT CCC AGT GTC C<br>Reverse: ATA CAA AGT TCC TGG AGA TGC
HPGD	Forward: TGG TCA ATA ATG CTG GAG TGA<br>Reverse: GGT TCC ACT GAT AAC AGA AAC CA
TXNIP	Forward: TCG GCT TTG AGC TTC CTC AG<br>Reverse: GTC TCT TGA GTT GGC TGG CT
GDF15	Forward: GTT GCA CTC CGA AGA CTC CAG<br>Reverse: GAG AGA TAC GCA GGT GCA GGT
TLE1	Forward: TGC TGT CCC TGA AAT TTG CT<br>Reverse: TGA CGA GGA CTC TTT GGA CT
ENC1	Forward: CAT CTC CCA CCT TGG TCC ACT<br>Reverse: CCA TGG CTC TGC TGA ACT GAA
RBM5	Forward: TAT GAT CCG ACA ACA GGG CTC<br>Reverse: AGC TGG CAC GTA GGT CTC TTT

Table S3. qRT-PCR primer sets used in this study (*continued*).

UGT2B15	Forward: GTG TTG GGA ATA TTA TGA CTA CAG TAA C<br>Reverse: GGG TAT GTT AAA TAG TTC AGC CAG T
UGT2B17	Forward: TGA CTT TTG GTT TCA AGC ATA<br>Reverse: TTC CAT TTC CTT AGG CAA GGG

Table S4. siRNA sequences used in this study.

HSP90α/β	Sense: AGU UUG AGA ACC UCU GCA AGC UCdA dT<br>Antisense: AUG AGC UUG CAG AGG UUC UCA AAC UUU
Non-specific (NS)	Sense: GUA CGG UUA UAC CGU GAC CdTdT<br>Antisense: GGU CAC GGU AUA ACC GUA CdTdT
